# Genome-wide identification analysis of the 4-Coumarate: CoA ligase (*4CL*) gene family expression profiles in *Juglans regia* and its wild relatives *J. Mandshurica* resistance and salt stress

**DOI:** 10.1186/s12870-024-04899-8

**Published:** 2024-03-23

**Authors:** Jiayu Ma, Dongjun Zuo, Xuedong Zhang, Haochen Li, Hang Ye, Nijing Zhang, Mengdi Li, Meng Dang, Fangdong Geng, Huijuan Zhou, Peng Zhao

**Affiliations:** 1 Key Laboratory of Resource Biology and Biotechnology in Western China, Ministry of Education, College of Life Sciences, Northwest University, Xi’an, Shaanxi 710069 China; 2 Xi’an Botanical Garden of Shaanxi Province, Institute of Botany of Shaanxi Province, Shaanxi Academy of Science, Xi’an, Shaanxi China; 3 College of Life and Environmental Sciences, University of Birmingham, Edgbaston, Birmingham, B15 2TT UK

**Keywords:** *4CL* gene family, *Juglans*, Anthracnose resistance, Salt stress

## Abstract

Persian walnut (*Juglans regia*) and Manchurian walnut (*Juglans mandshurica*) belong to Juglandaceae, which are vulnerable, temperate deciduous perennial trees with high economical, ecological, and industrial values. 4-Coumarate: CoA ligase (4CL) plays an essential function in plant development, growth, and stress. Walnut production is challenged by diverse stresses, such as salinity, drought, and diseases. However, the characteristics and expression levels of *4CL* gene family in *Juglans* species resistance and under salt stress are unknown. Here, we identified 36 *Jr4CL* genes and 31 *Jm4CL* genes, respectively. Based on phylogenetic relationship analysis, all *4CL* genes were divided into three branches. WGD was the major duplication mode for *4CL*s in two *Juglans* species. The phylogenic and collinearity analyses showed that the *4CL*s were relatively conserved during evolution, but the gene structures varied widely. 4CLs promoter region contained multiply *cis*-acting elements related to phytohormones and stress responses. We found that *Jr4CLs* may be participated in the regulation of resistance to anthracnose. The expression level and some physiological of *4CLs* were changed significantly after salt treatment. According to qRT-PCR results, positive regulation was found to be the main mode of regulation of *4CL* genes after salt stress. Overall, *J. mandshurica* outperformed *J. regia*. Therefore, *J. mandshurica* can be used as a walnut rootstock to improve salt tolerance. Our results provide new understanding the potential functions of *4CL* genes in stress tolerance, offer the theoretical genetic basis of walnut varieties adapted to salt stress, and provide an important reference for breeding cultivated walnuts for stress tolerance.

**Supplementary Information:**

The online version contains supplementary material available at 10.1186/s12870-024-04899-8.

## Introduction

Persian walnut (*Juglans regia*) and Manchurian walnut (*Juglans mandshurica*) belong to the *Juglans* genus in Juglandaceae [1]. Studies based on simplified genomes, chloroplast genomes, fossil evidence, and biogeographic historical reconstructions have found that these two walnut species diverged 20–31 million years ago [1–4]. *Juglans regia* (2n = 32), English walnut or Persian walnut, is an important oil-seeded perennial woody crop of monoeciousness and the oldest food source and widely cultivated nut-producing plant in the world [1, 5]. *J. regia* is originally from the mountains of Central Asia [6], it is an ancient cross between American and Asian lineages, dating back to the late 1900s [7]. It is distributed in Central, West and South Asia and Europe [1]. *J. regia* trees are light-loving and have strong resistance to drought, cold, and disease [1]. Its seeds are rich in a variety of nutrients needed by the human body, such as oil, vitamins, a variety of trace elements and minerals, etc., which have a certain therapeutic effect on slowing down the aging process and preventing heart disease and diabetes. It can also be made into biodiesel, edible walnut oil, and a variety of industrial chemicals, and is an important raw material for food and industrial applications [8–10]. *Juglans mandshurica* is a temperate deciduous tree whose wood and nuts are highly prized [11]. The fruit has a poor taste and is generally not eaten as a nut, but its seed kernel can be used as a traditional Chinese medicine to warm the kidney and moisten the intestines. In addition, tannin extracts are extracted from green husks, barks, and leaves, bark fibers are used as raw material for paper production. It grows mainly in the Asia region [11–14]. *J. mandshurica* as wild relative of *J. regia*, based on its potential as a reservoir of improved *J. regia* germplasm [11]. It is a hybrid breed with cultivated *J. regia* and can also be used as rootstock for *J. regia* because of its good resistance to stresses [11, 15, 16]. However, walnut production is challenged by environmental stresses, such as salinity, drought, and diseases [10, 17]. Recently, walnuts needed to improve the tolerance of different cultivars and varieties in its breeding program. Resistance studies of abiotic stresses in walnuts have focused on cold [18, 19] and drought tolerance [20, 21]. Mechanisms of salt resistance have been less well studied, whereas land salinization become more and more seriously during global climate changes. Therefore, to overcome the challenges of quality and quantity of walnuts, tolerance rootstocks is a fundamental strategy [22].

Salinity induces osmoregulation and increases antioxidant activities in plants [23, 24]. By applying appropriate stresses to plants, a range of physiological and morphological adaptations were activated, thereby increasing the plant’s tolerance to other stresses. Enhancement of antioxidant enzyme activities [25], antioxidant molecules [26], and photo stabilizers [27] under salt treatments were common responses in tolerating other environmental stresses, and thus the imposition of controlled salt stresses is expected to make the plant tolerant to various adverse environmental preparedness. Based on previous studies, moderate salt stress may induce multiple stress tolerance in *J. regia* [23]. There are several studies on germination, physiological, and antioxidative responses of salinity stress in *J. regia* [27, 28]. Recently, the chromosome-level genomes of *J. regia* [5] and *J. mandshurica* [11] have been published, which provides great significance for our comparative study of genome-wide differences of important gene families related to stress responses.

The phenylpropanoid metabolism pathway is essential for plant survival and provides plants with a large number of precursors for secondary metabolites, which contribute to growth and development and external environment stress resistance [29]. 4-Coumarate: CoA ligase (*4CL*) was first extracted by Mansell et al. [30] from cambial regions in *Salix* species. Because of this enzyme’s high catalytic specificity for 4-Coumaric acid, it is called 4-Coumarate: CoA ligase. The 4CL have a key role in linking lignin precursors to various other branching pathways and is one of the important enzymes in the phenylpropanoid metabolism pathway. *4CL* members affect plant growth, biosynthesis of phenylpropane derivatives, and environmental stress response, and can effectively regulate and improve plant-environment interactions. Current research suggested that several conserved polypeptide sequences were presented in 4CL protein sequences, including BOX I (SSGTTGLPKGV) and BOX II (GEICIRG) [31]. Among them, BOX I is highly conserved at the N-terminus and is the structural domain that binds to Adenosine monophosphate (AMP) and can directly participate in catalytic reactions [31]. BOX II is not directly involved in catalytic reactions, but the deficiency of this conserved sequence results in an almost complete loss of 4CL enzyme activity [32, 33]. Based on the functions of the proteins encoded by the *4CL* genes, *4CL* can be classified into three types, of which class I is mainly regulated in the biosynthesis of lignin compounds in plants, class II is mainly regulated the formation of flavonoid compounds, and class III is 4CL-like, whose specific function is still unclear [34].

The *4CL* genes have been extensively genome-wide identification studied in plant species, with 13 *At4CL* genes identified in *Arabidopsis thaliana*, 14 *Os4CL* genes in rice [35], 29 *Pbr4CL* genes in *Pyrus bretschneideri* [36], 35 *Eu4CL* genes in *Eucommia ulmoides* [37], 12 *Pg4CL* genes in *Punica granatum* [38] and 12 *Md4CL* genes in apple [39]. Since *4CL* genes are generally regulated in the process of plant response stresses, the studies of the regulatory mechanism and expression level of *4CL* genes are important for plant molecular biology [40–42]. The *Gh4CL7*-silencing cotton showed more sensitivity to drought treatment, while overexpressed *Gh4CL7 Arabidopsis* lines were more tolerant to drought treatment [40]. Overexpressed *Fm4CL2* was more tolerant to drought in tobacco [41]. Similarly, overexpression of *Fm4CL-like1* improved drought resistance in tobacco [43]. White light, UV irradiation, exogenous ABA, and PEG treatments promoted the up-regulation of the expression of *St4CL6* and *St4CL8* and suppressed the expression of *St4CL5*, which was hypothesized to promote the accumulation of lignin and flavonoids and improve the ability to scavenge ROS through increased gene expression, thus improving the ability of potato to resist abiotic stresses [42]. The expression of the *Pn4CL*s increased with time after infestation with *Phytophthora capsica* and peaked after 24 h [44]. Uhlmann et al. infected potato leaves with *Phytophthora infestans* and then 4CL enzyme activity increased 2-fold 12 h after inoculation [45]. Moreover, after inoculation with *P. capsica*, the expression levels of *Pn4CLs* were all higher in resistance than in susceptible. *Pn4CLs* were involved in the process of pepper resistance to *P. capsica* [46]. Infection of *Arabidopsis* with spores of *Peronospora parasitica* strongly induced *At4CL1* and *At4CL2* mRNA expression [46]. Despite a large number of previous studies, the regulatory mechanisms of *4CL* in response to stresses remain unclear. Identifying the *4CL* gene family and investigating the gene expression of *4CL* genes under stresses in two *Juglans* species may supply clues for further research on the gene function of *4CL* in perennial trees.

In our study, we first systematically identified two *Juglans 4CL* gene families. Then, we analyzed the characterizations of *4CLs*, including locations, collinearity relationships, *cis*-acting elements prediction, and microRNA target predictions. We also performed the gene expressions of *4CL*s using transcriptomic data from multiple organs and biotic stress. Subsequently, the performance of two *Juglans* species and the expression of *4CLs* under salt stress by physiological activity assays and qRT-PCR experiments. All the results provide the basis for the function of *4CLs* in two *Juglans* species, as well as clues on the regulations of *4CLs* in other woody plants.

## Materials and methods

### Morphology of *Juglans regia* and *Juglans mandshurica*

**Fig. 1:**
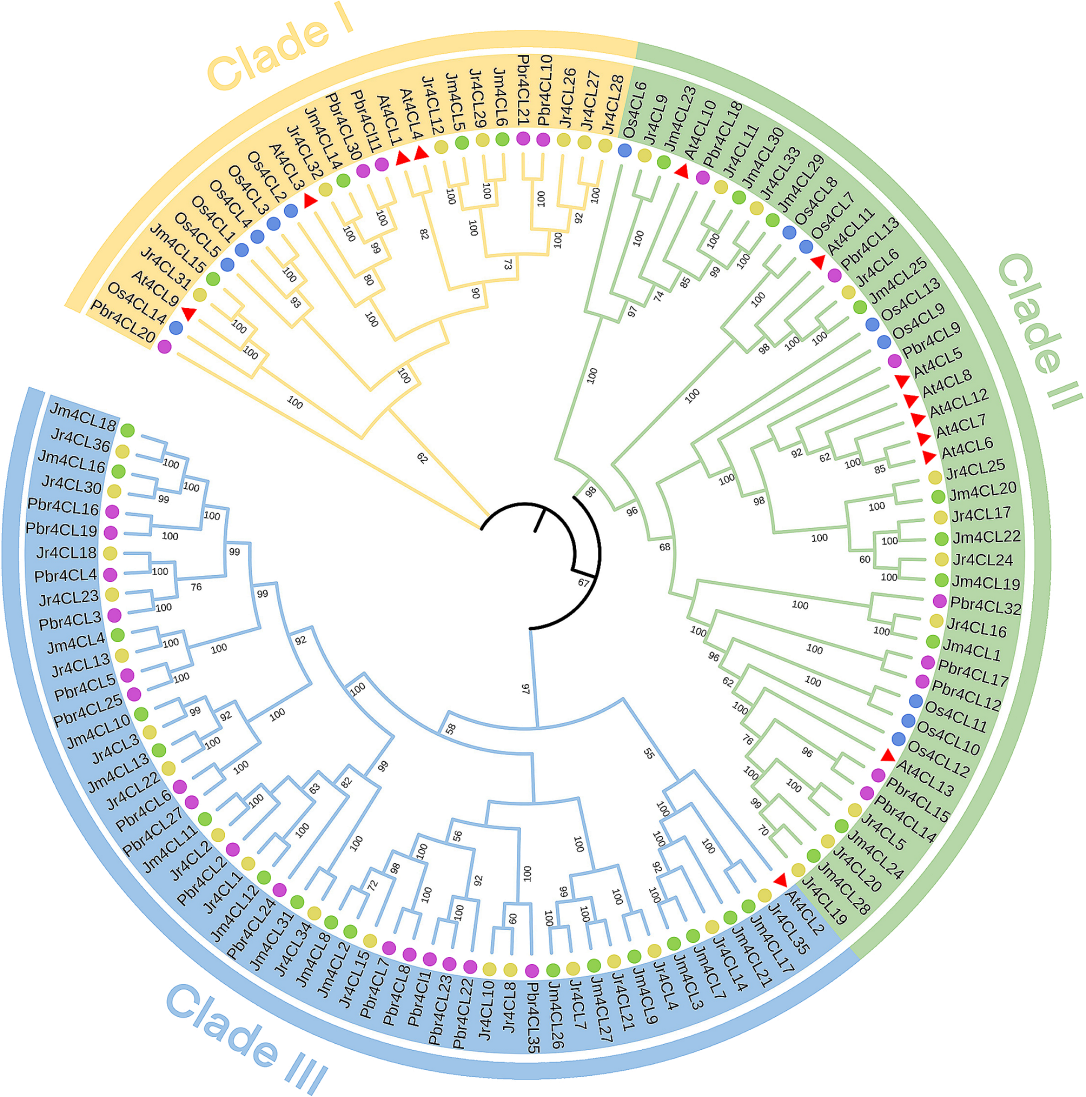
The phylogenetic tree of 4CL protein of *Arabidopsis thaliana*, *Oryza sativa, Pyrus bretschneideri, Juglans regia*, and *J. mandshurica*. The red triangles, bule, purple, yellow, and green circles represent *Arabidopsis*, rice, *P. bretschneideri*, *J. regia*, and *J. mandshurica*, respectively

In this study, we used the Persian walnut (*Juglans regia*) and Manchurian walnut (*Juglans mandshurica*) as plant materials (Fig. S1). The female flowers of *J. regia* are yellow (Fig. S1A) and the male flowers are catkins (Fig. S1B). The leaves are simple pinnately compound, compound leaves alternate, elliptic in shape with smooth margins (Fig. S1C). The fruit is oblate-globose (Fig. S1D). The female flowers of *J. mandshurica* are purplish red or bright red (Fig. S1E) and the male flowers are catkins (Fig. S1F). Leaves are simple pinnately compound, petiole is very short, the leaf shape is oblong, the leaf margin and serrulate, abaxial surface is densely pilose (Fig. 1G). The fruit is ovate or ovoid, with an acute tip, and the infructescence is usually 4-7-fruited (Fig. 1H).

### Genome-wide identification of *4CL* genes in *J. Regia* and *J. Mandshurica*

To identify 4CL candidate members in *J. regia* [5] and *J. mandshurica* [11], we used the proteins as query sequences of 13 At4CL members were downloaded from TAIR database (https://www.arabidopsis.org/). We performed the genome-wide identification using BLASTP (E-value < 1E^− 5^). To determine whether the 4CL candidate members involved the *4CL* gene family, the protein domains of these candidate members were queried in the NCBI CDD [47], Pfam [48], and SMART databases [49], and then the results were selected using excel. Candidate members containing the 4CL domain and AFD_class_I superfamily domain in CDD database, AMP-binding domain and AMP-binding_C domain in Pfam database, and 4CL domain and AFD_CAR_like domain in SMART database were the final members of the *4CL* gene family.

### Chromosomal localization and collinearity analysis

Chromosomal localization was visualized for all identified two *Juglans* species *4CL* genes based on gene annotation information using TBTOOLS software [50]. To facilitate subsequent studies, *4CL* genes were renamed according to the order of their position on the chromosome. Analysis of the 4CL genes collinearity between two *Juglans* species and three other selected species (*Arabidopsis*, *O. sativa*, and *P. bretschneideri*) [35, 36], as well as prediction of gene duplication events, was particular using MCScanX software [51]. The non-synonymous substitution (Ka), synonymous substitution (Ks) values, and the ratio of Ka/Ks in homologous genes were calculated using KaKs_Calclator v2.0 software [52].

### Physicochemical analyses and *Cis*-acting element prediction

Physicochemical properties and subcellular localization were analyzed on the ExPASy website [53], and WoLF PSORT website (https://wolfpsort.hgc.jp/), respectively. The *cis*-acting elements of the promoter region were predicted from all identified 2000 bp upstream sequences of the *4CL* genes using the PlantCARE online website [54]. Furthermore, the eggNOG-mapper online website (http://eggnog-mapper.embl.de/) was utilized to the identified *4CL* genes for GO (Gene Ontology) enrichment analysis [55].

### Phylogenetic and characteristic analysis

The 4CL protein sequences of five species (*J. regia, J. mandshurica, P. bretschneideri*, *Arabidopsis*, and *O. sativa*) [11, 35, 36] were constructed as a phylogenetic tree using MEGA v11 software (Maximum Likelihood method; bootstrap: 1000) [56]. We aligned the multiple sequences using ClustalX [57]. Beautification using the online website iTOL [58]. Based on the information provided by the CDD database in NCBI [47], using TBTOOLS [50] to show the distribution of characteristics domains on 4CL proteins of two walnut species. Analyzing the gene structure using the GSDS online website [59].

### Protein-protein interaction analysis and microRNA target prediction

We followed the protein-protein interaction analysis in STRING database (http://string-db.org) using all identified 4CL members’ protein sequences as query sequences and the *Arabidopsis* protein database as a reference. Visualization using Cytoscape software default parameters [60]. MicroRNA targeting prediction for all identified *4CL* members using default parameters from the psRNATarget online website [61].

### Plant material collection, treatment, and physiological indicators measurement

We collected the mature leaves of two *Juglans* species’ experimental materials. The mature leaves from two *Juglans* species were picked in the middle of July from Qinling National Forest Park in Shaanxi province [62]. Professor Peng Zhao identified *J. mandshurica* and *J. regia* based on the botanical characteristics of the leaves. We obtained permission to collect those plant samples from Qinling National Forest Park in Shannxi province. Salt stress treatments by soaking leaves in 150 mmol/L Na_2_SO_4_ solution. Removed the leaves after 24 h of salt treatment and soaked in clean water as a control. The obtained leaves were observed phenotypically. Then they were snap-frozen in liquid nitrogen and stored at -80 °C for backup. The voucher specimens of *J. regia* and *J. mandshurica* (deposition accession numbers: NWU2022024 and NWU2022025) were stored at the Evolutionary Botany Laboratory, College of Life Sciences, Northwest University (Xi’an, Shaanxi, China).

Subsequently, we determined the enzyme activities of two *Juglans* species salt-treatment and control groups leaves spectrophotometrically as follows. First, we took fresh leaves and added them to the extract for ice bath homogenization, centrifuged at 8000 g 4℃ and supernatant was taken. Then, superoxide dismutase (SOD), peroxidase (POD), and catalase (CAT) were determined by the spectrophotometric method using SOD, POD, and CAT assay kits (MolFarming, Nanjing China). All physiological indices were measured in three replicates of 0.1–0.2 g of fresh leaves. The mixture containing 0.3 mL of 260 mM methionine, 0.3 mL of 100 µM EDTA-Na2, 0.3 mL of 750 µM NBT, 0.3 mL of 20 µM riboflavin, 1.8 mL of variable-volume phosphate buffer, and extract was assayed for SOD activity at 560 nm. The POD activity was determined by monitoring the absorbance value at 470 nm of 1 mL of extract dissolved in 3 mL of mixed solution (containing 28 µL guaiacol and 19 µL H_2_O_2_ per 50 mL). The CAT activity was determined by monitoring the absorbance value at 240 nm of 500 µL of extract mixed into a 2.5 mL mixture containing 2 mL phosphate buffer and 500 µL H_2_O_2_ [63].

### Gene expression of *4CLs*

We determined the gene expressions of *4CLs* in two *Juglans* species. The samples from our previous collection included different tissues/organs (leaves, green husks, female and male flowers) [62, 64, 65]. All 24 different tissue/organ samples were sequenced by the Illumina HiSeq X Ten platform (Illumina, San Diego, CA, USA). Then, map all transcriptome clean reads to the reference genome using HISAT2 software [66]. Gene expression levels were calculated using fragments per kb of transcript sequence per million bp sequenced (FPKM) [67]. Calculate FPKM values using FeatureCounts software [68]. Sequencing of walnut fruit disease resistance data was downloaded from the public NCBI database [69]. These contain anthracnose-resistant varieties (F26) and anthracnose-susceptible varieties (F423). The K-means clustering was used to normalize *Jr4CL* genes fruit disease resistance data [70]. The gene expression level heatmaps were drawn using TBtools software [50].

Subsequently, the gene expression of the identified *4CL* genes under salt stress was further explored by qRT-PCR experiments. Each treatment has 3 biological replicates. Total RNA was isolated from leaves of two *Juglans* species using a plant RNA isolation kit (OMEGA, USA). Quality assessment of total RNA based on A260/A280 ratio using the Nanodrop spectrometer (KAIAO, Beijing, China). Reverse transcription to complementary DNA (cDNA) using quality-tested RNA. cDNA template was obtained by reverse transcription using 5× PrimeScript RT Master Mix (Takara) reverse transcriptase. We diluted the cDNA as 5-fold as the template DNA for qRT-PCR experiment. Using 2×Plus SYBR real-time PCR mixture (Biotec) as fluorescent dye, a mixture containing 10 µL 2×plus SYBR real-time PCR mixture, 0.5 µL Primer F, 0.5 µL Primer R, 2 µL cDNA, and 7 µL nuclease-free water was used for qRT-PCR experiments on Bio-Rad CFX96 fluorescence quantitative PCR instrument [62]. The reaction procedure was pre-denaturation at 94 °C for 2 min, denaturation at 94 °C for 15 s, annealing at 58 °C for 15 s, extension at 72 °C for 30 s, and 40 cycles. The reaction was subsequently terminated by plate reading at 95 °C for 5 s after 65 °C dissolution curve capture. *J. regia* β-actin was used as an internal reference gene [62, 71] and the primers were designed with the online Primer3Plus website (https://www.primer3plus.com). The relative expression of all *4CLs* was normalized by the 2^-ΔΔCT^ method [72]. All the primer sequences are shown in Table S1.

## Results

### Identification and phylogenetic relationship of *4CL* genes in *J. regia* and its wild relatives *J. mandshurica*

We identified total of 36 *Jr4CL* genes and 31 *Jm4CL* genes (Table S2). To facilitate subsequent studies, we renamed all genes according to their position order on the chromosome. Table S2 listed the baseline information for all identified *4CL* gene members.

The maximum likelihood phylogenetic tree was performed using 4CL protein sequences of *Arabidopsis* (13), *O. sativa* (14), *P. bretschneideri* (29), *J. regia* (36), and *J. mandshurica* (31). Then, we classified their evolutionary relationships based on the phylogenetic tree (Fig. 1). All 4CL gene members were divided into three clades (Clade I, II, and III). The largest clade is Clade III, which contained 1 *At4CL*, 18 *Jr4CLs*, 17 *Jm4CLs*, and 16 *Pbr4CLs*, followed by the Clade II, which contained 8 *At4CLs*, 8 *Os4CLs*, 8 *Pbr4CLs*, 11 *Jr4CLs*, and 10 *Jm4CLs*. The remaining *4CL* members were assigned to Clade I, which contained 4 *At4CLs*, 6 *Os4CLs*, 5 *Pbr4CLs*, 7 *Jr4CLs*, and 4 *Jm4CLs*. All four species were distributed in all branches except *Os4CL*, which was distributed in only two branches (Clade I and II). In Clade I-III, three woody plants (*J. regia*, *J. mandshurica*, and *P. bretschneideri*) were clustered, such as (1) *Jm4CL15* and *Jr4CL31*; (2) *Pbr4CL11, Pbr4CL30, Jm4CL14*, and *Jr4CL32*; (3) *Jr4CL12, Jm4CL5, Jr4CL29*, and *Jm4CL6* were clustered in the same branch in Clade I. (4) *Pbr4CL32*, *Jr4CL16* and *Jm4CL1*; (5) *Jr4CL25*, *Jm4CL20, Jr4CL17, Jm4CL22, Jr4CL24*, and *Jm4CL19* were clustered in the same branch in Clade II. (6) *Pbr4CL35, Jr4CL8*, and *Jr4CL10*; (7) *Pbr4CL2, Jr4CL2*, and *Jm4CL11* were clustered in the same branch in Clade III. These results suggested that three woody plants are more closely related.

### Physicochemical analyses and subcellular localization prediction of 4CL members

We predicted the physicochemical properties of the 4CL proteins of the two *Juglans* species with the following results (Table 1). The mean length of Jr4CL protein ranged from 523 aa (Jr4CL4) to 694 aa (Jr4CL14), with a mean length of 564 aa. The mean length of Jm4CLs was consistent with that in Jr4CLs, but ranged more widely, from 307 aa (Jm4CL31) to 1083 aa (Jm4CL28). The molecular weights of Jr4CLs varied from 56369.53 kDa (Jr4CL21) to 76612.12 kDa (Jr4CL14) with a mean molecular weight of 61718.09 kDa. The molecular weights of Jm4CLs were greater than those of Jr4CLs, which varied from 34330.35 kDa (Jm4CL31) to 117466.84 (Jm4CL28), with a mean of 61800.35 kDa. In addition, there are 23 and 16 acidic proteins (isoelectric point less than 7) in Jr4CLs (Jr4CL1, Jr4CL, Jr4CL4, Jr4CL6, Jr4CL7, Jr4CL12, Jr4CL13, Jr4CL15, Jr4CL16, Jr4CL18, Jr4CL19, Jr4CL20, Jr4CL21, Jr4CL26, Jr4CL27, Jr4CL28, Jr4CL29, Jr4CL30, Jr4CL31, Jr4CL32, Jr4CL34, Jr4CL35, and Jr4CL36) and Jm4CLs (Jm4CL2, Jm4CL5, Jm4CL6, Jm4CL8, Jm4CL9, Jm4CL14, Jm4CL15, Jm4CL16, Jm4CL17, Jm4CL21, Jm4CL24, Jm4CL25, Jm4CL26, Jm4CL27, Jm4CL28, and Jm4CL31), respectively. It was also found that the Jr4CLs and Jm4CLs clustered in Clade I were all acidic proteins (Fig. 1). Most of the members of Jr4CLs and Jm4CLs had instability indices less than 40 (26 of 36 for Jr4CL and 21 of 31 for Jm4CL), and the total mean hydrophilicity of 18 Jr4CLs (50%) and 21 Jm4CLs (67.7%) was negative, so most of the Jm4CLs were considered to be stable hydrophilic proteins. Additionally, most of the identified 4CL members were located on the plasma membrane, which may be related to the function of 4CL proteins (Table 1).

**Table 1:** The predicated protein information of 4CL in *Juglans regia* and *J. mandshurica*

Gene name	No. of amino acids	Mol. Wt (Da)	Isoelectric point (pI)	Instability index (II)	Aliphatic index	Grand average of hydropathicity (GRAVY)	Subcellular localization^a^
Jr4CL1	553	60808.00	6.95	42.81	90.72	-0.058	pero
Jr4CL2	555	60992.05	6.83	38.14	86.00	-0.087	pero
Jr4CL3	582	64270.92	7.60	34.50	90.62	-0.136	pero
Jr4CL4	523	56425.31	5.72	34.81	94.21	-0.032	plas
Jr4CL5	561	61038.41	8.19	46.91	99.73	0.051	plas
Jr4CL6	567	62023.66	6.25	35.81	97.94	0.127	plas
Jr4CL7	524	56455.41	6.36	37.83	92.75	-0.014	plas
Jr4CL8	577	63187.75	8.94	43.78	91.82	-0.054	plas
Jr4CL9	542	59471.00	8.73	41.64	97.08	0.034	plas
Jr4CL10	564	62168.99	7.00	43.88	99.40	0.077	plas
Jr4CL11	540	59266.45	8.22	38.75	102.11	0.071	plas
Jr4CL12	544	59499.91	6.06	41.19	96.76	-0.013	chlo
Jr4CL13	565	61938.44	6.79	35.70	92.53	-0.027	pero
Jr4CL14	694	76612.12	7.17	31.63	89.88	-0.083	chlo
Jr4CL15	570	62344.13	5.49	37.67	90.00	-0.125	chlo
Jr4CL16	573	62260.32	5.86	41.61	97.03	-0.020	nucl
Jr4CL17	549	59487.76	8.67	37.30	102.46	0.095	plas
Jr4CL18	590	65202.60	6.80	33.51	82.10	-0.155	chlo
Jr4CL19	557	60340.56	6.25	45.30	97.67	0.069	plas
Jr4CL20	558	60322.60	6.71	42.86	97.87	0.085	E.R.
Jr4CL21	525	56369.53	6.22	34.54	96.82	0.015	plas
Jr4CL22	587	65052.87	8.17	32.67	91.70	-0.147	pero
Jr4CL23	563	61944.21	8.39	32.91	83.85	-0.132	pero
Jr4CL24	550	59855.19	8.63	34.35	99.07	0.044	plas
Jr4CL25	577	63203.98	9.05	38.56	96.46	-0.044	plas
Jr4CL26	550	60633.02	5.95	32.01	98.05	-0.013	chlo
Jr4CL27	543	59784.21	5.54	31.36	99.50	-0.026	chlo
Jr4CL28	542	59606.04	5.61	30.37	99.52	0.001	chlo
Jr4CL29	545	59330.79	5.69	34.70	100.35	0.040	chlo
Jr4CL30	574	62588.95	5.42	33.74	93.97	0.014	chlo
Jr4CL31	558	61012.74	5.81	36.82	97.96	0.018	cyto
Jr4CL32	572	62323.73	5.54	38.05	99.14	0.070	plas
Jr4CL33	544	59498.00	8.80	39.12	100.48	0.079	plas
Jr4CL34	545	60320.58	6.12	34.17	97.06	0.077	cyto
Jr4CL35	661	73839.49	5.98	40.94	84.66	-0.167	nucl
Jr4CL36	573	62371.41	6.86	34.48	97.89	0.084	chlo
Jm4CL1	603	65862.31	7.74	44.42	93.81	-0.130	nucl
Jm4CL2	589	64468.68	6.28	34.78	88.78	-0.206	chlo
Jm4CL3	685	76413.19	8.33	36.05	89.78	-0.077	chlo
Jm4CL4	536	59092.98	9.23	40.47	95.00	0.037	pero
Jm4CL5	544	59472.84	5.87	41.12	96.05	-0.008	chlo
Jm4CL6	540	58924.38	6.50	34.84	98.93	-0.002	chlo
Jm4CL7	582	64207.82	8.51	29.47	90.96	-0.062	chlo
Jm4CL8	568	62025.13	6.62	33.32	85.67	-0.169	chlo
Jm4CL9	523	56473.35	5.75	35.19	93.84	-0.049	plas
Jm4CL10	428	47362.40	7.60	36.43	90.23	-0.164	pero
Jm4CL11	399	44252.13	8.08	42.10	89.37	-0.027	pero
Jm4CL12	396	43952.83	8.53	41.52	90.08	-0.059	pero
Jm4CL13	587	64958.71	7.61	32.69	91.70	-0.143	pero
Jm4CL14	523	56955.42	5.39	37.61	97.99	0.079	plas
Jm4CL15	558	60958.67	5.75	35.88	98.49	0.024	cyto
Jm4CL16	558	61082.29	6.65	30.31	90.34	-0.063	chlo
Jm4CL17	661	73931.66	5.86	41.91	84.66	-0.176	nucl
Jm4CL18	537	58783.23	9.08	37.37	94.47	-0.012	chlo
Jm4CL19	539	58507.49	8.09	37.78	98.94	0.073	plas
Jm4CL20	577	63220.02	9.05	38.36	97.14	-0.033	plas
Jm4CL21	698	76909.30	6.68	37.26	90.64	-0.070	chlo
Jm4CL22	549	59439.76	8.53	38.35	103.53	0.113	plas
Jm4CL23	590	65296.44	8.78	37.21	97.31	0.003	plas
Jm4CL24	530	57363.95	5.92	43.57	100.06	0.097	plas
Jm4CL25	573	62693.39	6.11	37.66	98.27	0.132	plas
Jm4CL26	524	56484.37	6.33	37.26	90.88	-0.029	plas
Jm4CL27	527	56712.87	6.22	34.77	96.26	-0.002	plas
Jm4CL28	1083	117466.84	6.06	44.90	93.01	0.012	plas
Jm4CL29	669	73445.78	8.53	38.44	98.16	-0.008	plas
Jm4CL30	497	54762.36	9.03	40.10	100.97	0.046	E.R.
Jm4CL31	307	34330.35	5.55	46.49	94.92	-0.013	cyto

^a^*Note* pero: peroxisome; plas: plasma membrane; chlo: chloroplast; nucl: nucleus; E.R.: endopiasmic reticulum; cyto: cytoskeleton

### Chromosomal localization and duplication mode of *4CL*s

**Fig. 2:**
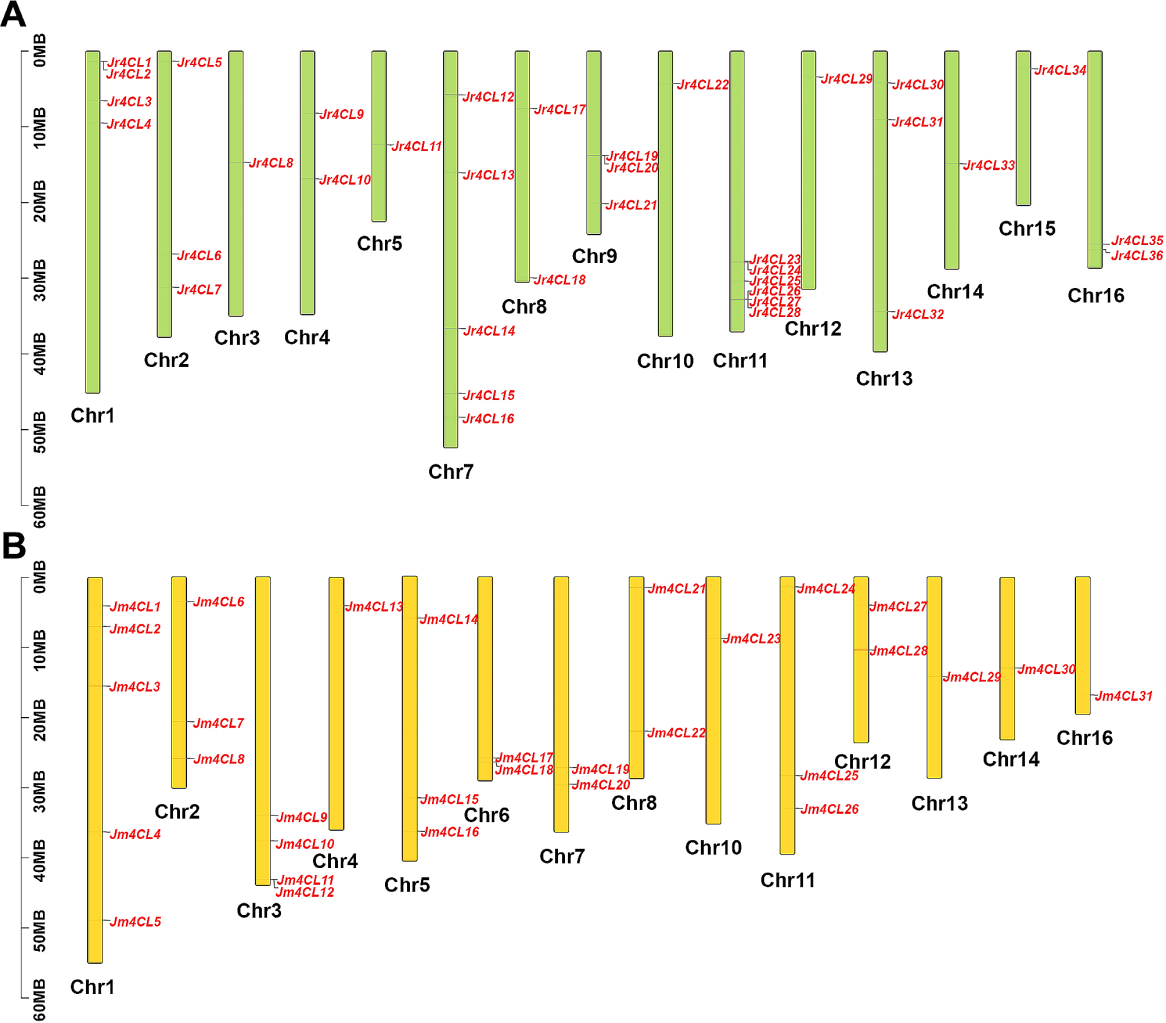
The locations of *Jr4CL* genes (A) and *Jm4CL* genes (B)

For *J. regia*, 36 *4CLs* were randomly and irregularly located in 15 different chromosomes, except for chromosome 6 (Fig. 2A). Chromosome 11 had the highest *Jr4CL* density of 16.67%, followed by chromosome 7 with *Jr4CL* density of 13.89%. Next was chromosome 1, with *Jr4CL* density of 11.11%. Chromosome 2, chromosome 9, and chromosome 13 all contained 3 *Jr4CLs* with a gene density of 8.33%. Chromosome 4, chromosome 8, and chromosome 16 contained 2 *Jr4CLs* with a gene density of 5.56%, respectively. For *J. mandshurica*, 31 *Jm4CLs* were distributed on 14 different chromosomes, with the exception of chromosomes 9 and 15 (Fig. 2B). Chromosome 1 had the largest *Jm4CL* gene density of 16.13%, followed by chromosome 3 with a gene density of 12.90%. Chromosome 2, chromosome 5, and chromosome 11 contained 3 *Jm4CLs* with a gene density of 9.68%. Chromosome 6, chromosome 7, chromosome 8, and chromosome 12 contained 2 *Jm4CLs* with a gene density of 6.45%. The other *4CL*s were situated on distinct chromosomes.

To investigate the reasons for the expansion of the *Jr4CL* and *Jm4CL* gene family, we analyzed the duplication patterns of the *4CL* genes. Gene duplication pattern of the *4CL* genes contained four models, consisting of whole genome duplication (WGD), tandem duplication (TD), proximal duplication (PD), and dispersed duplication (DSD; Table S3) [73]. We found that WGD was the predominant model in both *Juglans* species. WGD duplication patterns accounted for 17 of the 36 *Jr4CLs* (47.22%) and 20 of the 31*Jm4CLs* (64.52%). Nine *4CL*genes in both *J. regia* (*Jr4CL6, Jr4CL8, Jr4CL10, Jr4CL13, Jr4CL15, Jr4CL16, Jr4CL32, Jr4CL34, and Jr4CL35*) and *J. mandshurica* (*Jm4CL1, Jm4CL4, Jm4CL14, Jm4CL15, Jm4CL17, Jm4CL20, Jm4CL25, Jm4CL30, Jm4CL31*) were experienced DSD, respectively. Both *4CL* genes in *Jr4CLs* (*Jr4CL26* and *Jr4CL27*) and *Jm4CLs* (*Jm4CL11* and *Jm4CL12*) underwent TD, respectively. There were 3 *Jr4CLs* (*Jr4CL1, Jr4CL2*, and *Jr4CL28*) experienced PD. Notably, the *Jr4CL9, Jr4CL18, Jr4CL20, Jr4CL25*, and *Jr4CL31* were identified as singleton and did not experience any of the above four duplication patterns.

### Conserved domains and gene structures of 4CL members

**Fig. 3:**
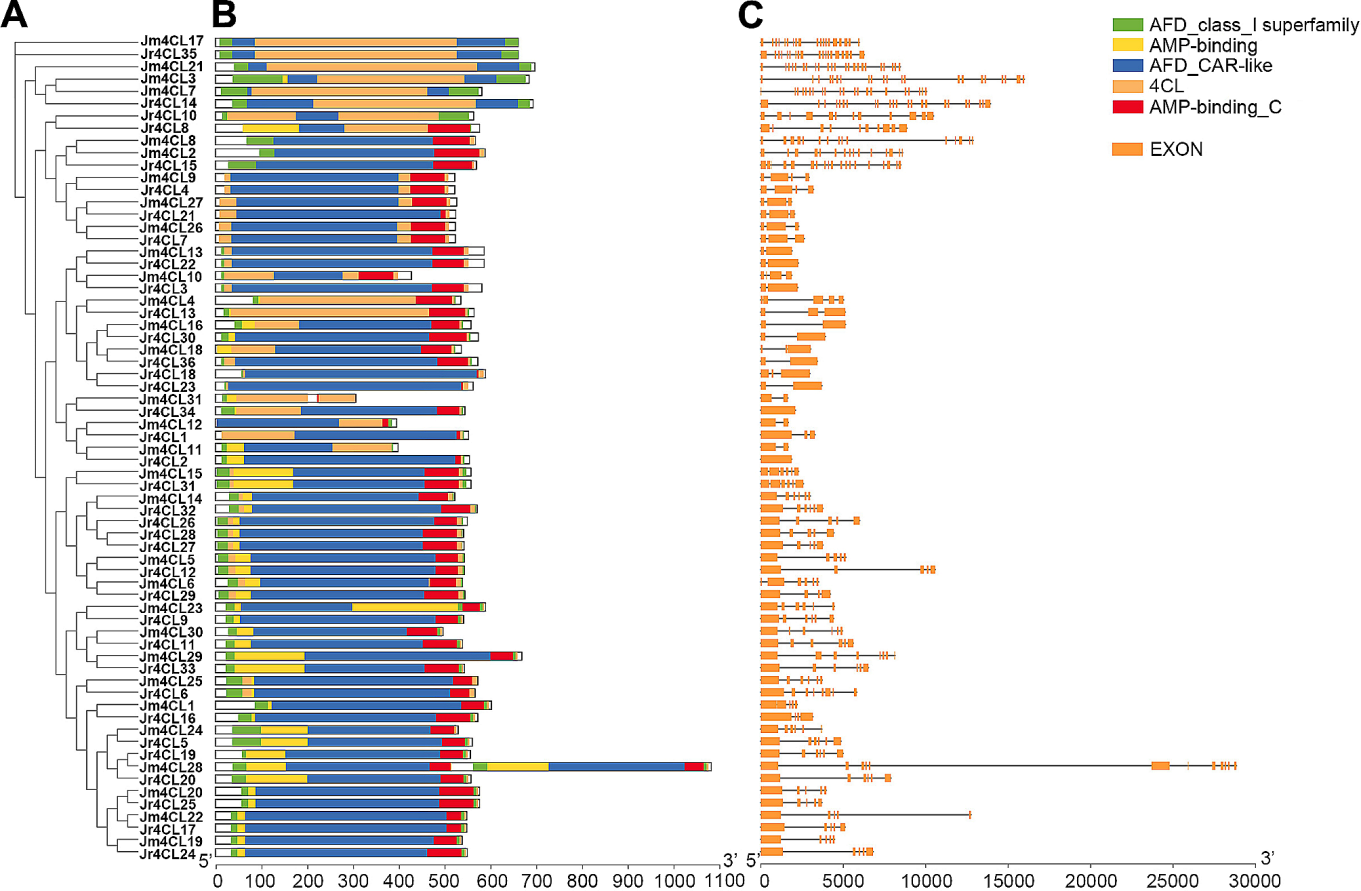
The characterization of 4CL members. (A) Phylogenetic tree of *4CLs* in two *Juglans* species; (B) Conserved domains of 4CL proteins. (C) Gene structures of *4CLs*. Orange boxes and gray lines indicate exons and introns, respectively

The results of the maximum likelihood tree were constructed based on two *Juglans* species (Fig. 3A) similar to the phylogenetic tree clustering results constructed using five selected species (Fig. 1). The results indicated that the structural domains of the *4CL* gene family members were conserved to a high degree. All identified 4CL proteins contained 4CL domain and conserved kinase structural domains (Fig. 3B). Sequence alignment also suggested that the 4CL proteins of two *Juglans* species were highly conserved in BOX I (SSGTTGLPKGV) and BOX II (GEICIRG) regions (Fig. S2).

Variations of gene structure may affect differences in gene function, so we recognized the gene structure of all identified *4CL*s (Fig. 3C; Table S4). The gene structure results showed that all identified *4CL* genes differed significantly in their structures. The exon count of *Jr4CLs* was from 1 to 23, and the exon count of *Jm4CLs* was from 2 to 23. Among them, 9 *Jr4CLs* contained 6 exons, followed by 7 *Jr4CLs* contained 5 exons and 5 *Jr4CLs* contained 2 exons. In *J. mandshurica*, 7 *Jm4CLs* contained 6 exons, 6 *Jm4CLs* contained 5 exons, and 5 *Jm4CLs* contained 3 exons. *Jr4CL14* and *Jm4CL21* contained the highest number of exons at 23. Notably, individual *4CL* genes contain longer introns, especially Jm*4CL28*.

### Synteny analysis of *4CL* genes

**Fig. 4:**
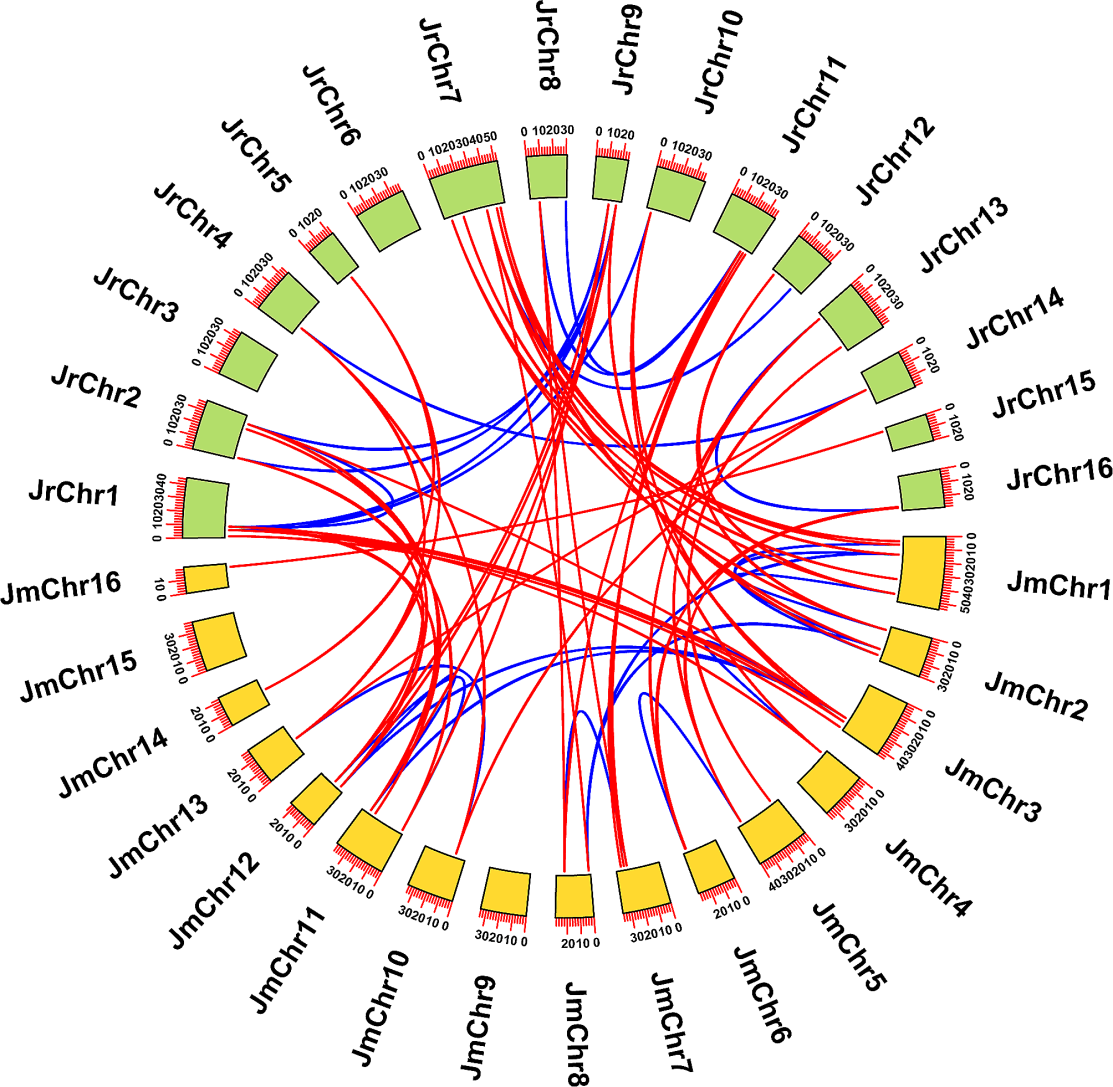
Collinearity analysis for *4CL* genes among two *Juglans* species. Red and blue lines indicate orthologous and paralogous gene pairs, respectively

The collinearity analysis revealed 11 and 15 paralogous gene pairs in *Jr4CLs* and *Jm4CLs*, respectively (Fig. 4; Table S5). There were a total of 48 *4CL* orthologous gene pairs between *Jr4CLs* and *Jm4CLs* (Fig. 4; Table S5). The number of orthologous gene pairs was much greater than the number of paralogous gene pairs, suggesting a high degree of collinearity in *Jr4CLs* and *Jm4CLs*. Moreover, in two *Juglans* species 8 *Jr4CLs* (*Jr4CL8, Jr4CL10, Jr4CL14, Jr4CL20, Jr4CL23, Jr4CL26, Jr4CL27, Jr4CL28, Jr4CL31*) and 3 *Jm4CLs* (*Jm4CL1, Jm4CL12, Jm4CL15*) without paralogous gene pairs, suggest that they may be *J. regia* or *J. mandshurica* specific genes that did not collinearity. To expose the selection pressure among *4CL* homologous gene pairs, we computed Ka and Ks parameters (Table S5). The Ka/Ks ratios of all *4CL* homologous gene pairs were smaller than 1, indicating that all gene pairs underwent purifying selection and may have evolved relatively slowly.

Subsequently, to investigate the possible evolutionary processes of *4CL*s, we performed collinearity analyses between two *Juglans* species and three selected plants, including one monocotyledon (*O. sativa*) and two dicotyledon species (*Arabidopsis* and *P. bretschneideri*). The *Jr4CLs* and *Jm4CLs* have 20 and 19 orthologous gene pairs with *Arabidopsis*, respectively (Fig. S3A; Table S6). Jr4CLs and Jm4CLs have 8 and 4 orthologous gene pairs with *O. sativa* (Fig. S3B; Table S7), and have 26 and 26 orthologous gene pairs with *P. bretschneideri*, respectively (Fig. S3C; Table S8). In our present study, the number of *4CL* homologous gene pairs was higher in two *Juglans* species and dicotyledon plants than with monocotyledon plants. While in both dicotyledon species, the number of *4CL* homologous gene pairs was higher in two *Juglans* species and *P. bretschneideri* than *Arabidopsis*, suggesting that two *Juglans* species are more closely evolutionarily related to *P. bretschneider* than to *Arabidopsis*. Notably, 2 *Jr4CLs* (*Jr4CL5* and *Jr4CL13*) and 2 *Jm4CLs (Jm4CL4*and *Jm4CL24*) had homologous gene pairs with all three selected species, suggesting that these 4 homologous gene pairs may have existed before the differentiation of monocotyledon and dicotyledon plants.

### Analysis of *cis*-acting elements and GO annotation for *4CLs*

**Fig. 5:**
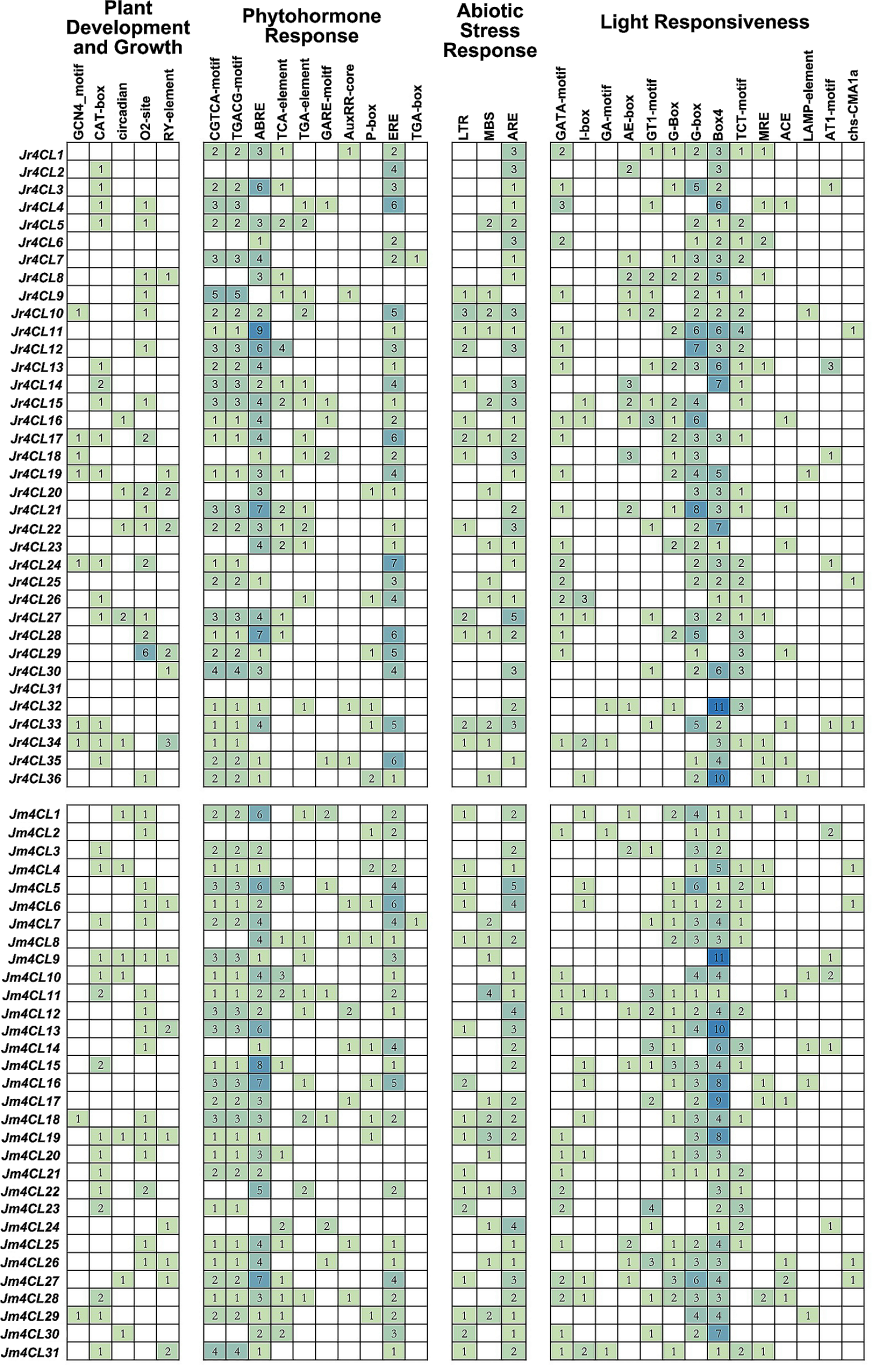
The analysis of *Cis*-acting elements. The colored numbers indicate the number of *cis*-acting elements

To understand the potential function of the *4CL* genes in *J. regia* and *J. mandshurica*, we analyzed *cis*-acting elements of the promoter regions and GO functional annotation (Fig. 5). The *cis*-acting elements of the promoter regions were divided into four categories, including plant development and growth, phytohormone response, abiotic stress response, and light responsiveness (Fig. 5). Overall, a greater number of *cis*-acting elements were found in *Jr4CLs* than in *Jm4CLs*, suggesting that *Jr4CLs* may be involved in more complex signal transduction pathways. Most of the identified *4CL* genes contain *cis*-acting elements associated with light responsiveness, and the *Jr4CLs* promoter regions had more *cis*-acting elements responsive to light than *Jm4CLs*, suggesting that *Jr4CLs* may be more sensitive to light. It is noticed that we found a significant proportion of identified *4CL* members respond to three *cis*-acting elements associated with Methyl Jasmonate (MeJA) hormone response and Abscisic Acid (ABA) hormone response. In addition, the promoter regions of *4CL cis*-acting elements were mostly associated with abiotic stresses, suggesting that they might be important in abiotic stress resistance of *Jr4CLs* and *Jm4CLs*, especially in anaerobic induction (LTR).

The GO enrichment analysis of all identified *4CL* members showed that the fatty acid biosynthetic process, fatty acid metabolic process, jasmonic acid biosynthetic process, small molecule metabolic process, cellular lipid metabolic process, and ketone biosynthetic process were the top six BPs (Biological processes) subgroups in *J. regia* (Fig. S4A). The microbody and peroxisome were the top two cellular components (CCs) subgroups, while the fatty acid ligase activity and acid-thiol ligase activity were the top two molecular functions (MFs) subgroups (Fig. S4A). For *J. mandshurica*, the jasmonic acid biosynthetic process, jasmonic acid metabolic process, carboxylic acid metabolic process, small molecule metabolic process, and cellular lipid metabolic process were the top five BPs subgroups, while the microbody and peroxisome were the top two CC subgroups. The top three MF subgroups include the CoA-ligase activity, acid-thiol ligase activity, and ligase activity, forming carbon-sulfur bonds (Fig. S4B).

### Prediction of protein-protein interaction and miRNAs targeting of 4CL members

**Fig. 6:**
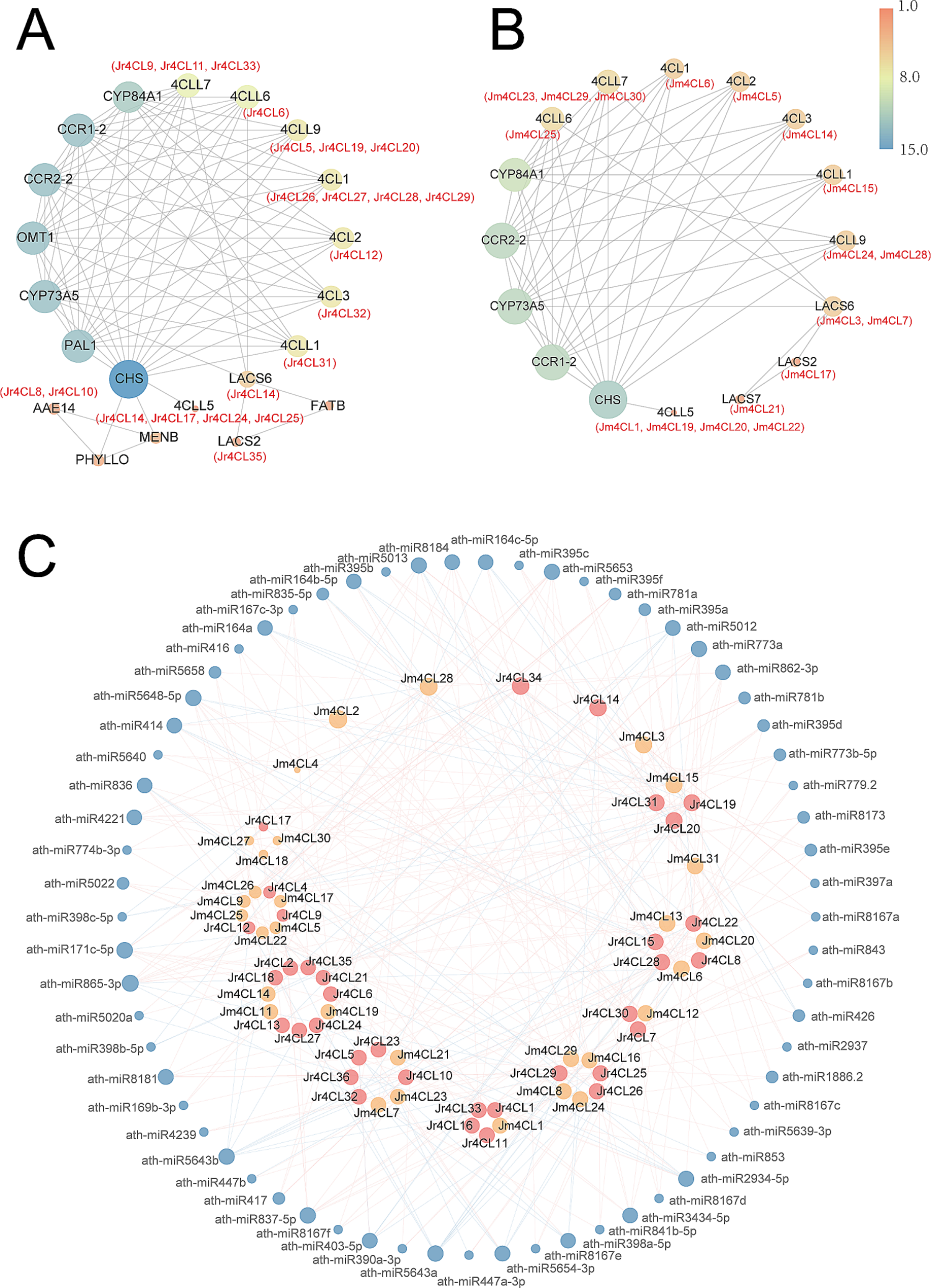
Protein-protein interaction and microRNA target of 4CL members. (A) Protein-protein interaction of the Jr4CL proteins; (B) Protein-protein interaction of the Jm4CL proteins. Colored circles represent proteins, grey lines indicate interaction; (C) MicroRNA targeting of the *4CL* genes in two *Juglans* species. The blue circle, red circle, and orange circle represent microRNAs, *Jr4CLs*, and *Jm4CLs*, respectively. The size of the circle represents how much of the targeting relationship. Blue lines represent translation and red lines represent cleavage

We used the homology mapping method to predict the interactions of 4CL proteins in two *Juglans* species based on the interactions of 4CL proteins in *Arabidopsis* (Table S9). As shown in Fig. 6A, B, both Jr4CLs and Jm4CLs mapped to 11 *Arabidopsis* proteins, and all identified 4CL proteins mainly interacted with CHS proteins. In addition, compared to Jm4CLs, Jr4CLs interacted with more proteins, suggesting that Jr4CLs were involved in more complex signaling pathways.

A total of 260 microRNAs were predicted to target 35 *Jr4CLs* (except *Jr4CL3*) and 218 microRNAs were predicted to target 30 *Jm4CLs* (except *Jm4CL10*, Fig. 6C, Table S10). Among them, 356 miRNAs modulated the expression of identified *4CL*s by cleavage, and 122 miRNAs modulated by translation (Fig. S4). Notably, *Jr4CL34* and *Jm4CL2* were the target genes of 15 and 19 different miRNAs, respectively, and were the genes with the highest number of miRNA targets. In addition, some miRNAs target different *4CL* genes, such as the ath-miR865-3p targets 12 different *4CL* genes at the same time.

### Gene expression levels of *Jr4CLs* and *Jm4CLs*

**Fig. 7:**
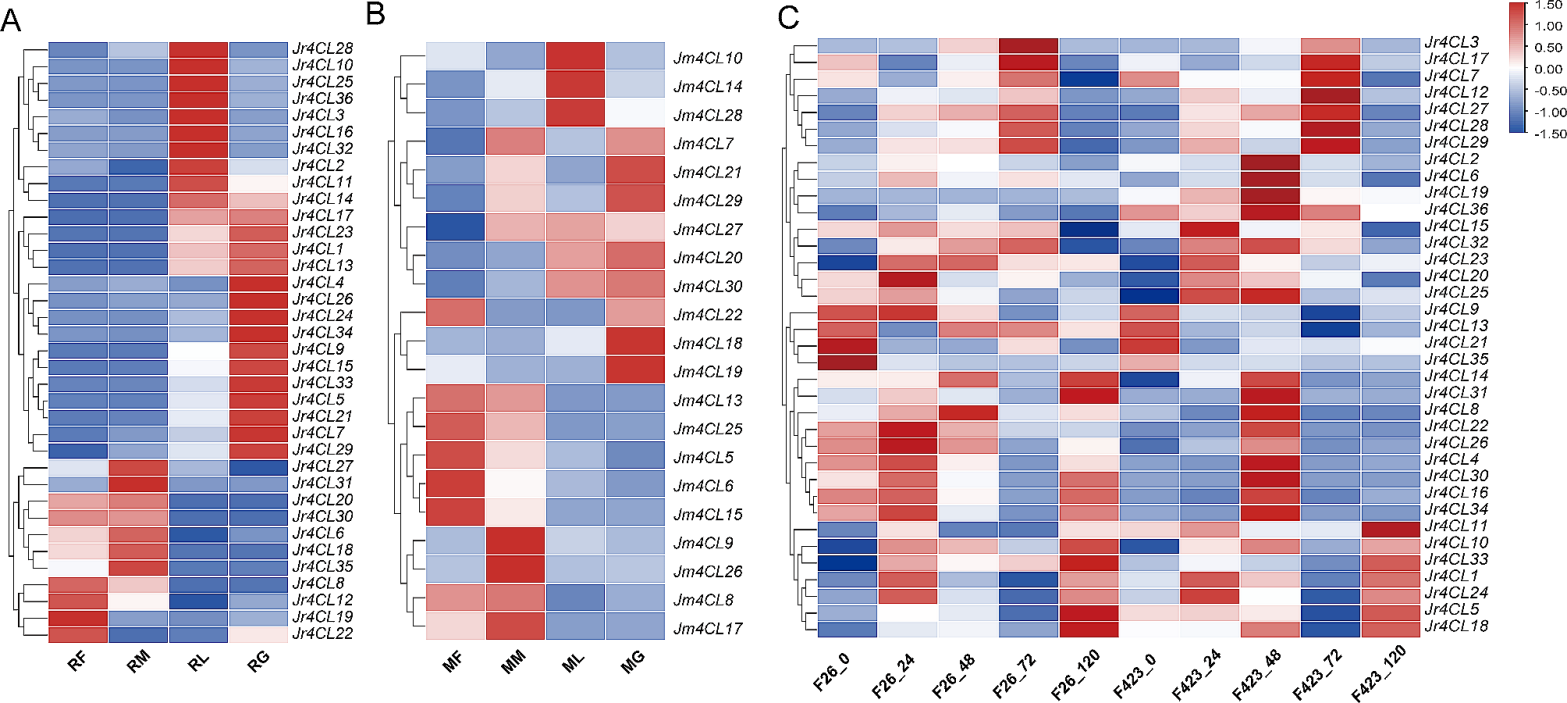
Gene expression levels of *4CLs*. (A) *Jr4CLs* and (B) *Jm4CLs* in different organs; (C) Gene expression profiles of *Jr4CLs* under biotic stress

To investigate the expression pattern of *4CL* genes, we analyzed the expression of all identified *4CL*s in four selected organs, including leaves, green husks, male, and female flowers based on transcriptomic data (Fig. 7A and B). According to the transcriptome results, all the identified 36 *Jr4CL*s were expressed in four selected organs. Among them, 14 *Jr4CLs* were highly expressed in leaves, *17 Jr4CLs* in green husks, 8 *Jr4CLs* in female flowers, and *8 Jr4CLs* in male flowers, respectively (Fig. 7A; Table S11). However, for *J. mandshurica*, only 21 *Jm4CLs* were expressed in four selected organs. Of these, there were 6 *Jm4CLs* expressed highly in leaves, 9 *Jm4CLs* in green husks, 8 *Jm4CLs* in male flowers, and 12 *Jm4CLs* in female flowers, respectively (Fig. 7B; Table S12). The different expression patterns in two *Juglans* species suggested that all *Jr4CLs* have roles in the growth and development of the selected organs, while *Jm4CLs* may not function in some organs of *J. mandshurica*. Additionally, 6 *Jr4CLs* and 7 *Jm4CLs* were only expressed at high levels in female or male flowers (Fig. 7). It is suggested that these genes might be related to the heterodichogamous.

### Genes expression patterns of *Jr4CLs* under biotic stress

To explore the role of *Jr4CLs* in biotic stress (disease) response, we examined the transcriptome gene expression levels of different walnut varieties (F26 and F423) under biotic stress (Fig. 7C; Table S13). Overall, F26 had higher levels of *4CL* gene expression than F423, suggesting that *Jr4CLs* may function in *J. regia* anthracnose resistance. *Jr4CL9* and *Jr4CL13* expression gradually decreased with time after infection with F423 varieties, whereas expression increased with time after infection with F26 varieties, which suggests that they could be associated with the resistance to anthracnose in walnuts.

Based on K-means clustering analysis, the expression trends of both F26 and F423 were classified into 9 subgroups (Fig. S6; Table S14). Most of the *Jr4CLs* showed a gradual increase in expression over 24 h in F26 varieties, reached peak expression at 24 h, followed by a gradual decrease, and then increased again after 72 h (Fig. S5A). In contrast, in the F423 variety, most of the *Jr4CLs* reached the highest expression at 48 h, followed by a gradual decrease (Fig. S5B).

### Genes expression and antioxidant enzyme activity changes of *4CLs* under salt stress

**Fig. 8:**
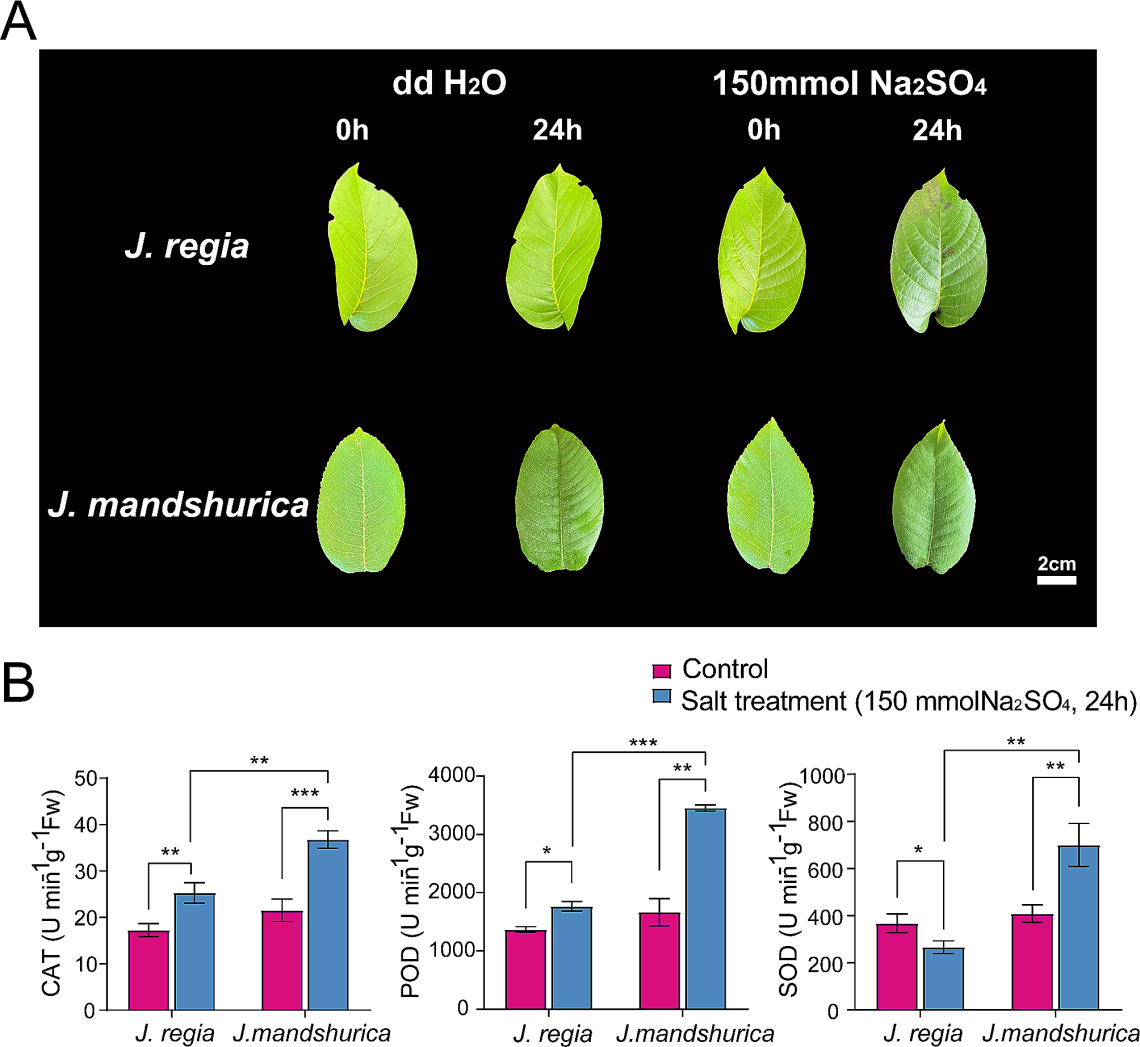
Characteristics of two *Juglans* species leaves under salt stress. (A) Phenotypes of *J. regia* and *J. mandshurica* leaves under salt stress, where ddH_2_O_2_ indicated control group, 150 mmol/L Na_2_SO_4_ indicated salt-treated group; (B) Antioxidant enzyme activities of *J. regia* and *J. mandshurica* leaves under salt stress. CAT: Catalase, POD: Peroxidase, SOD: Superoxide dismutase. The red and blue columns indicated the control and salt-treated groups, respectively. The statistical significance using t-test. *=*p* < 0.05, **=*p* < 0.01, ***=*p* < 0.001

To better study the differences between two *Juglans* species under salt stress, we separately salt-treated them at the same developmental period leaves and assayed the enzyme activities (Fig. 8). Based on the phenotype results, it can be seen that both *Juglans* species leaves were damaged to varying degrees after salt treatment as compared to the control (Fig. 8A). There were visible black patches on the *J. regia* leaves, and the *J. mandshurica* leaves were significantly wrinkled although did not show black patches compared to those before the salt treatment. Compared to the control, CAT activity increased 1.464 and 1.707 times, and POD activity increased 1.288 and 2.077 times after salt treatment in *J. regia* and *J. mandshurica* leaves, respectively. However, SOD activity decreased 1.383 times in *J. regia* and increased 1.715 times in *J. mandshurica* leaves after salt treatment (Fig. 8B). In addition, the levels of all three enzyme activities were higher in *J. mandshurica* leaves than in *J. regia* leaves. All these results indicate that *J. mandshurica* performed better than *J. regia* under salt stress.

**Fig. 9:**
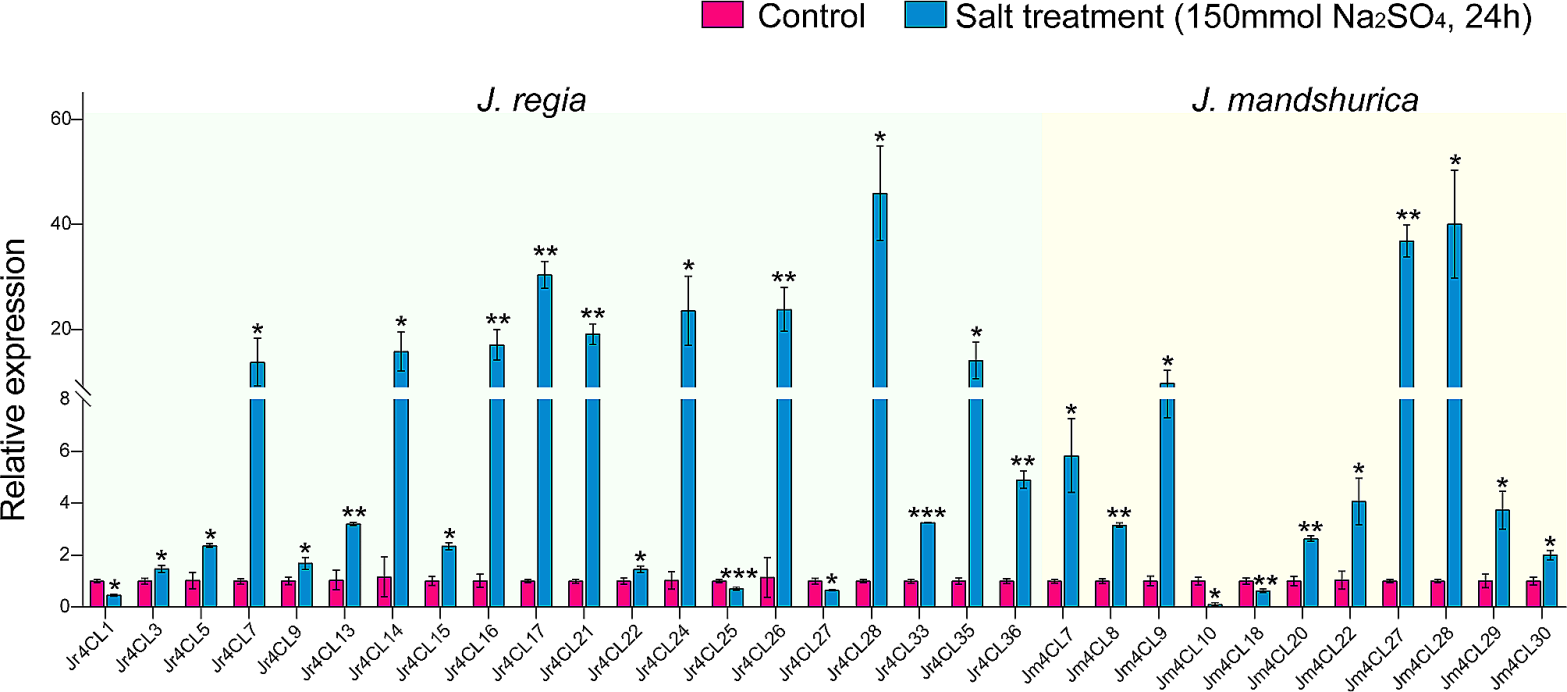
The qRT-PCR experiments of *4CLs* in *J. regia* and *J. mandshurica* leaves under salt stress. The red and blue columns represent the control and salt-treated groups, respectively. The control group was treated with ddH_2_O_2_ and the salt-treated group was treated with 150 mmol/L Na_2_SO_4_ in two *Juglans* species leaves. The green and yellow backgrounds represent *J. regia* and *J. mandshurica*, respectively. The statistical significance using t-test. *=*p* < 0.05, **=*p* < 0.01, ***=*p* < 0.001

To better understand the potential roles of these genes in salt tolerance in two *Juglans* species, we further analyzed the *4CL* gene expression levels in leaves under salt stress using qRT-PCR (Fig. 9; Fig. S7). There were 28 *Jr4CLs* (except *Jr4CL4*, *Jr4CL8*, *Jr4CL18*, *Jr4CL19*, *Jr4CL29*, *Jr4CL30*, *Jr4CL31*, and *Jr4CL34*) and 19 *Jm4CLs* (except *Jm4CL1*, *Jm4CL2*, *Jm4CL3*, *Jm4CL4*, *Jm4CL11*, *Jm4CL12*, *Jm4CL13*, *Jm4CL15*, *Jm4CL16*, *Jm4CL23*, *Jm4CL24*, and *Jm4CL31*) expressed in leaves, respectively (Fig. 7A and B; Table S11; S12). Among the expressed 28 *Jr4CL* genes, total of 21 *Jr4CLs* (75%) increased the expression level in leaves after salt treatment leaves, while 7 *Jr4CL* genes (25%) decreased the expression level. Among the 19 *Jm4CLs*, 15 *Jm4CLs* (78.95%) increased in expression and 4 *Jm4CLs* (21.05%) decreased in expression after salt treatment. These results suggest that *Jr4CLs* and *Jm4CLs* may respond to salt stress through two different modes (positive and negative) of regulation, of which positive regulation may be predominant. Notably, the expression levels of some *4CL* genes increased dramatically after salt treatment. For example, *Jr4CL17* increased by 30.4 times, *Jr4CL28* increased by 44.6 times, *Jm4CL27* increased by 36.84 times, and *Jm4CL28* increased by 41.299 times, respectively. It is suggested that these *4CLs* may function in *4CL* gene resistance to salt stress.

## Discussion

Two *Juglans* species were both economically and ecologically important woody tree species. Recently, the comparative genomic studies of *Juglans* species have become a research hotspot [9, 11, 74]. 4CL is one of the key enzymes in phenylpropanoid metabolism pathway and the final step in phenylpropanoid synthesis pathway [75]. The level of 4CL enzyme activity had a significant impact on the accumulation of compounds, such as flavonoids and lignin. Furthermore, *4CLs* have key roles in plant growth and resistance to environmental stresses from outside [76]. We identified a total of 36 and 31 *4CL* gene members in *J. regia* and *J. mandshurica*, respectively. The large variation in the number of *4CL* genes in different species [35–39] may be related to the gene duplication events experienced by different species during evolution. WGD is the most common gene duplication pattern, and WGD events may enhance the adaptability of species to their environment [77]. WGD events occurred in most of all identified *4CLs*, of these 47.22% in *Jr4CLs* and 64.52% in *Jm4CLs*. This suggests that WGD events play an important role in duplication events of the *4CL*gene family in two *Juglans* species (Table S3).

According to their phylogenetic relationships, all identified *4CL* genes can be divided into three subgroups (Clade I-III), and *4CLs* of three woody plants were clustered, indicating that the three woody plants are more closely related to each other (Fig. 1). Most *Jr4CLs* and *Jm4CLs* were distributed in the plasma membrane and chloroplast (Table 1). Similarly, *Gh4CLs* were widely distributed [40], but *Cit4CLs* were only localized in the cytoplasm [78], which may be related to the different locations where the *4CLs* function. *Jr4CLs* and *Jm4CLs* were unevenly distributed on the chromosome, but some *4CL*s had high similarity and collinearity, such as Jr4CL1 and Jr4CL2, Jr4CL19 and Jr4CL20, Jm4CL11 and Jm4CL12 (Figs. 2 and 4). Collinearity results showed that 6 *Jr4CLs* were not collinear with any of the other four species, suggesting that they may be unique genes in *J. regia* (Fig. 4; Fig. S2; Table S5-8). Whereas *Jm4CLs* were collinear with the other four selected species, indicating that *Jm4CLs* may be relatively conserved over the course of evolution. All *Jr4CLs* and *Jm4CLs* homologous gene pairs Ka/Ks were smaller than 1, which shows that all gene pairs underwent purifying selection during evolution (Table S5). These results suggesting that the functions of *4CLs* may have been conserved over the course of evolution. The structural domains of 4CL proteins were highly conserved in two *Juglans* species (Fig. 3B), while all identified 4CL proteins contained conserved Box I and Box II sequences in multiple sequence analysis (Fig. S2), which is consistent with the results of previous studies [31]. However, the gene structures of *4CLs* showed large differences in two *Juglans* species, with exon numbers ranging from 1 to 23 (Fig. 3C; Table S4). The same phenomenons were observed in most species of *4CL* gene families. For example, the number of exons in *Md4CL* [39] and *Eu4CL* gene families [37] also ranged from 1 to 23. An increment in the number of introns in genes may be better than harm to the plant. As non-coding regions, introns could protect genes from mutations and thus better preserve gene function [79, 80]. *Cis*-acting elements are involved in the regulation of gene expression, and a variety of *cis*-acting elements in gene promoters may be associated with different gene functions [64]. A significant population of identified *4CL*s contained *cis*-acting elements in response to hormones such as MeJA, ABA, and abiotic stresses such as anaerobic induction (Fig. 5), indicating that *Jr4CLs* and *Jm4CLs* may be involved in the regulations of MeJA, ABA and anaerobic induction in plants.

Protein-protein interaction prediction results showed that most 4CL proteins had interactions with CHS proteins (Fig. 6A and B). 4CL catalyzes the formation of 4-coumaroyl CoA, which subsequently generates chalcone by chalcone synthase (CHS), the first important enzyme in flavonoid pathway [81]. The phenylpropane metabolic synthesis pathway is active in a wide range of plants when exposed to environmental stresses, providing precursors for the synthesis of flavonoids, lignins, phenolic acids, etc. The research showed that CHS activity was enhanced after exposure to exogenous pathogenic microorganisms [82], suggesting that 4CL may interact with CHS and play a role in defense against plant pathogens. 4CL proteins and CHS proteins co-regulated the production of flavonoid metabolites that regulate seed germination [83] and improve plant tolerance and resistance to adversity stresses [84]. MicroRNAs have become a research hotspot because they regulate gene transcription and thus affect plant productivity in many processes such as growth, development, and environmental stresses [85]. MiRNAs adaptive responses can increase plant survival in environments such as drought, salinity, and pathogens [86]. We found that 35 *Jr4CLs* and 30 *Jm4CLs* were predicted to be the target genes of 260 and 218 *Arabidopsis* miRNAs, respectively (Fig. 6C; Table S10). Among them, cleavage was the main mode of miRNA regulation of *4CL* gene expression.

The expression of *4CL*s in plants is specific in their different tissues/organs, with *At4CL1* and *At4CL2* being the most strongly expressed in seedling roots, while *At4CL3* had high expression levels in flowers [75]. In pomegranate, *Pg4CL1, Pg4CL4, Pg4CL5, Pg4CL6*, and *Pg4CL11* were highly expressed in roots, leaves, flowers, and pericarps. While *Pg4CL7* was highly expressed only in leaf and exocarp [38]. In cotton, different *4CLs* also showed different expression patterns in different organs [40]. *Gh4CL2, Gh4CL15, Gh4CL17, Gh4CL19, Gh4CL23*, and *Gh4CL33* were highly expressed in stems, and *Gh4CL8, Gh4CL30*, and *Gh4CL34* were highly expressed in roots. *Euc4CL17, Euc4CL23, Euc4CL29*, and *Euc4CL31* were highly expressed only in male and female flowers of *Eucommia ulmoides* [37]. Similar phenomena were observed in two *Juglans* species. All *Jr4CLs* were expressed in four organs (Fig. 7A). Nevertheless, only 21 *Jm4CLs were* expressed in selected organs, and the other 10 *Jm4CLs* may function in other tissues or other developmental stages (Fig. 7B). *Juglans* species were heterodichogamous [87, 88]. Interestingly, six *Jr4CL* genes (Jr4CL12, Jr4CL19, Jr4CL22, Jr4CL27, Jr4CL31, and Jr4CL35) and seven *Jm4CL* genes (Jm4CL7, Jm4CL9, Jm4CL21, Jm4CL22, Jm4CL26, Jm4CL27, and Jm4CL29) were highly expressed only in male or female flowers (Fig. 7), suggesting that those *4CL* genes may be related to the heterodichogamous in walnut species, but the regulatory mechanisms involved remain to be further investigated.

Walnut anthracnose, caused by *Colletotrichum gloeosporioides*, is one of the most serious walnut diseases, causing early defoliation of walnut and resulting in reduced fruit production [89]. Chemical control has been the main measure for controlling walnut anthracnose, but it is limited by the pathogen’s resistance and the impact on environment, chemical control measures are limited [90–92]. Therefore, the breeding of disease-resistant varieties is of extreme interest. We explored the expression pattern of *Jr4CLs* in F26 and F423 (Fig. 7C; Table S13). We found that the expression level of *Jr4CLs* were generally increased in F26 over F423. Most *Jr4CLs* after infection with F26 showed an increase and then a decrease in expression levels, which increased again after 72 h. Expression levels of most *Jr4CLs* after infection with F423 increased over time at 48 h, and peaked at 48 h, followed by a gradual decrease in expression levels (Fig. S6). These results all suggest that *Jr4CLs* may function in *J. regia* resistance to anthracnose. In previous studies, *J. mandshurica* has better disease resistance than *J. regia*, and *J. mandshurica* is often used as a rootstock for *J. regia* to enhance its disease resistance [11]. Therefore, it is presumable that *Jm4CLs* may have better disease resistance performance compared to *Jr4CLs*, but further studies are needed.

Walnuts are less salt tolerant and more sensitive to salt [28]. After treatment of mature leaves of two *Juglans* species with 150 mmol/L Na_2_SO_4_ solution for 24 h, *J. mandshurica* had better performance than *J. regia* (Fig. 8A). Consistent with previous studies that concluded that *J. mandshurica* resistance is superior to *J. regia* [11, 14–16, 93, 94]. Under normal growth conditions, plants maintain a certain level of antioxidant enzyme system activity, scavenging the superoxide radicals that are constantly generated, so that the antioxidant enzyme activity and the superoxide radical content in the plant reach a certain equilibrium relationship. However, when plants are under various environmental stresses (such as drought, salt damage, extreme temperatures, pests, and diseases), it will lead to the production of a large number of reactive oxygen species (ROS), resulting in the impairment of the cellular structure and function, which will affect its growth and development, and even lead to its death [95, 96]. The accumulation of ROS induced by environmental stresses prompts plants to eliminate ROS by synthesizing antioxidant enzyme systems through signal transduction [96, 97]. SOD in the antioxidant system is the first line of defense to protect plant cells from oxygen free radicals and scavenge ROS [98]. POD is an endogenous scavenger of ROS in plants under environmental stresses and coordinates with SOD and CAT to scavenge excess free radicals in plants, maintain free radicals in plants at a normal level and enhance plant resilience [99]. When plants are in an adverse environment, antioxidant enzymes maintain high activity, which can keep free radicals and reactive oxygen species at relatively low levels and mitigate the effects on plant cells [100] Therefore, the level of antioxidant enzyme activity can reflect the strength of plant stress tolerance to a certain extent. After salt treatment, the determination of POD, SOD, and CAT activities in treated and control groups of *J. regia* and *J. mandshurica* leaves, to some extent could reflect the degree of response of antioxidant enzyme systems to salt stress in the two *Juglans* species subjected to salt stress. In the present study, the activities of all three antioxidant enzymes in *J. mandshurica* leaves increased significantly after salt treatment (Fig. 8B). However, the activities of CAT and POD in *J. regia* leaves increased significantly after salt treatment, whereas the activity of SOD decreased significantly. SOD is an important enzyme in the plant antioxidant system for scavenging free radicals, which breaks down superoxide anions and defends against cell membrane damage caused by ROS [101, 102], whereas J. regia leaves showed a decrease in SOD activity under salt stress, suggesting that damage caused by excess ROS could no longer be eliminated under this treatment condition (150 mmol/L Na_2_SO_4_, 24 h), this treatment condition may have reached the limit of *J. regia* leaves to resist salt damage. A similar phenomenon was observed in *Solidago canadensis* [103], where CAT and SOD activities increased and POD activity decreased after salt treatment. The CAT and POD contents of strawberries [104] showed a tendency to increase and then decrease with the duration of salt treatment. POD and SOD activities of *Betula platyphylla* increased significantly after salt treatment [105]. In ginger, POD, SOD, and CAT showed significant elevation after salt stress [106]. After salt stress, SOD, CAT, and POD gradually accumulated in salt-tolerant lines of asparagus with the rise of time, while the activities of the three enzymes decreased in salt-susceptible lines at the late stage of treatment [107]. These are the response of plants to protect their normal growth in extreme environments by increasing their antioxidant enzyme system. The increase in SOD, POD, and CAT activities under appropriate salt concentration stress is a self-protection of plants against unfavorable environments, but when there is an excessive accumulation of reactive oxygen radicals, the antioxidant enzymes are reduced by lipid peroxidation of plant cell membranes which makes the cell membranes unstable, and therefore there is a tendency for a decrease in the SOD activity of *J. regia* after salt stress, which is similar to the results of the previous studies [103–107]. In general, the overall levels of increase in activity of antioxidant enzymes in *J. mandshurica* leaves were higher than that of *J. regia*, indicating that *J. mandshurica* leaves have better salt tolerance than *J. regia*, in agreement with the phenotypic result.

Previous studies have shown that 4CL genes are capable of responding to plant adversity stress and that different 4CL gene family members of the same species may differ in their response to stress [108]. Moreover, *4CL*s expression levels were significantly changed under salt stress (Fig. 9; Fig. S7), demonstrated that *4CL* genes of two *Juglans* species may function in response to salt stress. And *4CL* genes may expressed in response to salt stress through positive or negative regulation, with positive regulation predominating. The expression trend of *4CLs* under salt stress was consistent, but the degree of change was different, and the change in the degree of response contributed to better and faster response of *4CL* genes to salt stress. Individual genes showed a sharp increase in expression levels after salt stress, showing that these *4CLs* function in two *Juglans* species’ resistance to salt stress. A similar phenomenon was observed in cotton, especially for *Gh4CL21, Gh4CL24, Gh4CL27*, and *Gh4CL31*, which showed a significant increase in expression levels after salt stress [40]. In *Eucommia ulmoides*, all 35 *Euc4CLs* responded to salt stress, and the expression levels of most of the *Euc4CLs* increased significantly after salt treatment, especially *Euc4CL9, Euc4CL17* and *Euc4CL27* [37]. In Mulberry, all four *Ma4CLs* responded to salt stress. All *Ma4CL1-3* showed an overall up-regulation under salt stress. While *Ma4CL4* showed a trend of up-regulation in stems and down-regulation in roots after salt stress [109]. 4CLs were regulated with salt resistance transcription factors such as MYB [110], bHLH [111], NAC [112], bZIP [113], and AP2/ERF [114], especially MYB (Fig. S8), suggesting that these 4CL genes are key candidates for salt tolerance. However, the functions of these *Jr4CLs* and *Jm4CLs* need to be further studied and verified. Walnuts are widely cultivated in Xinjiang Province in China, and central Asia, mostly growing in mountainous and saline areas [1, 5]. *J. mandshurica* has better salt tolerance than *J. regia*, so *J. mandshurica* can be used as a rootstock for walnuts to improve the salt tolerance of walnut fruit trees against the external environment and increase the fruiting rate of walnuts.

## Conclusion

In this study, we comprehensively genome-wide identified the *4CL* gene family members in both *Juglans* species. Phylogenetic analysis showed that the *4CL* genes were divided into three branches. Collinearity analysis showed that the *4CL* genes were relatively conserved during evolution, but the gene structures varied widely, which was similar to the results in other plants. Gene expression analysis showed that both *Jr4CLs* and *Jm4CLs* play key roles in the resistance of two *Juglans* species to resistance stresses. Under salt stress treatment, both phenotypic results and antioxidant enzyme activity analyses suggest that *J. mandshurica* had better performance than *J. regia*. Therefore, *J. mandshurica* can be used as a rootstock for *J. regia* to resist diseases and salt damage. Laying the theoretical foundation for walnut germplasm resource enhancement for salt stress. It is also necessary to further explore the unique roles of *Jr4CLs* and *Jm4CLs* in other stresses (e.g., plant nematode disease, extreme temperatures, drought, flooding, etc.).

### Electronic supplementary material

Below is the link to the electronic supplementary material.

Supplementary Material 1

Supplementary Material 2

## Data Availability

The raw data were downloaded from the NCBI Sequence Read Archive (SRA) database under accession number (GSE147083).

## Abbreviations

4CL: 4-Coumarate:CoA ligase
AMP: Adenosine monophosphate
POD: Peroxidase
SOD: Superoxide Dismutase
CAT: Catalase
WGD: Whole genome duplication
TD: Tandem duplication
PD: Proximal duplication
DSD: Dispersed duplication
MeJA: Methyl Jasmonate
ABA: Abscisic acid
CHS: Chalcone synthase
