# Supplementary material for: Genome-wide identification analysis of the 4-Coumarate: CoA ligase (4CL) gene family expression profiles in Juglans regia and its wild relatives J. Mandshurica resistance and salt stress
Source: BMC Plant Biol. 2024 Mar 23;24:211. doi: 10.1186/s12870-024-04899-8 (PMC10960452; doi:10.1186/s12870-024-04899-8)

**Additional File 1**

**Figure S1**: Morphological diagrams of *J. regia* and *J. mandshurica.* (A-D) *J. regia* female flowers, male flowers, leaf and fruits, respectively; (E-H) *J. mandshurica* female flowers, male flowers, leaf and fruits, respectively.


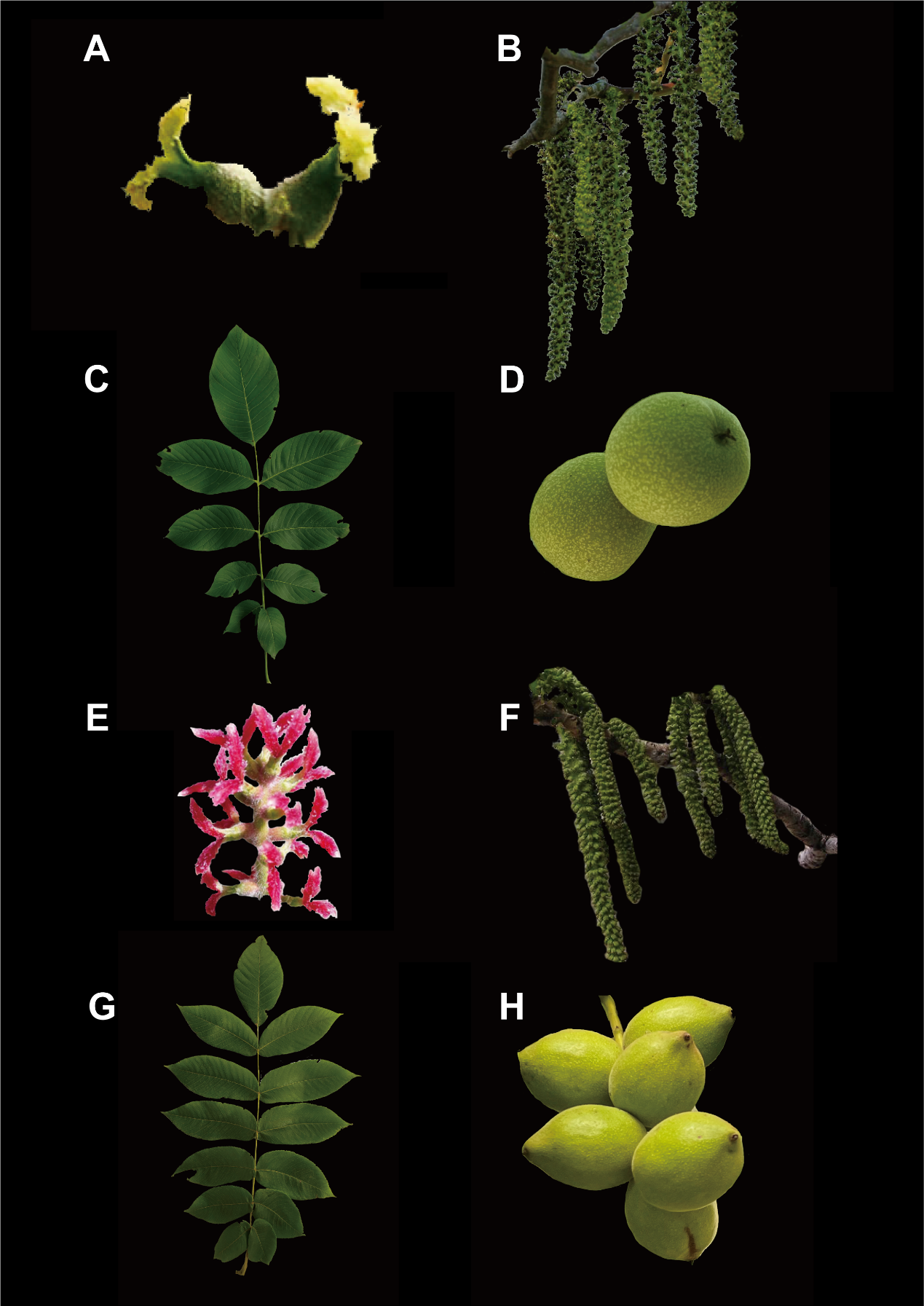


**Figure S2:** Alignment of the sequences of Jr4CL and Jm4CL proteins.


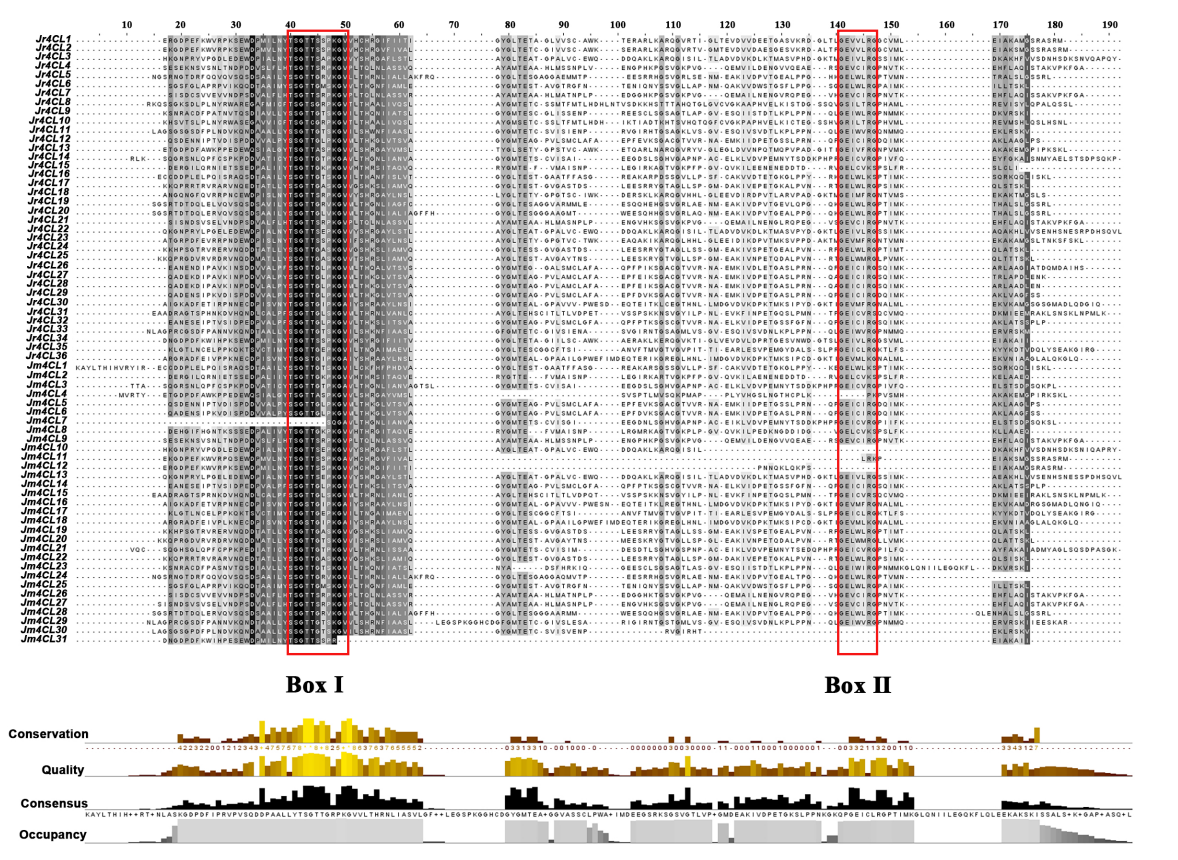


**Figure S3:** The collinearity relationships of *4CL* genes. (A) Collinearity relationships of *4CL* genes among two *Juglans* species and *Arabidopsis*; (B) Collinearity relationships of *4CL* genes among two *Juglans* species and rice; (C) Collinearity relationships of *4CL* genes among two *Juglans* species and *P. bretschneideri*.


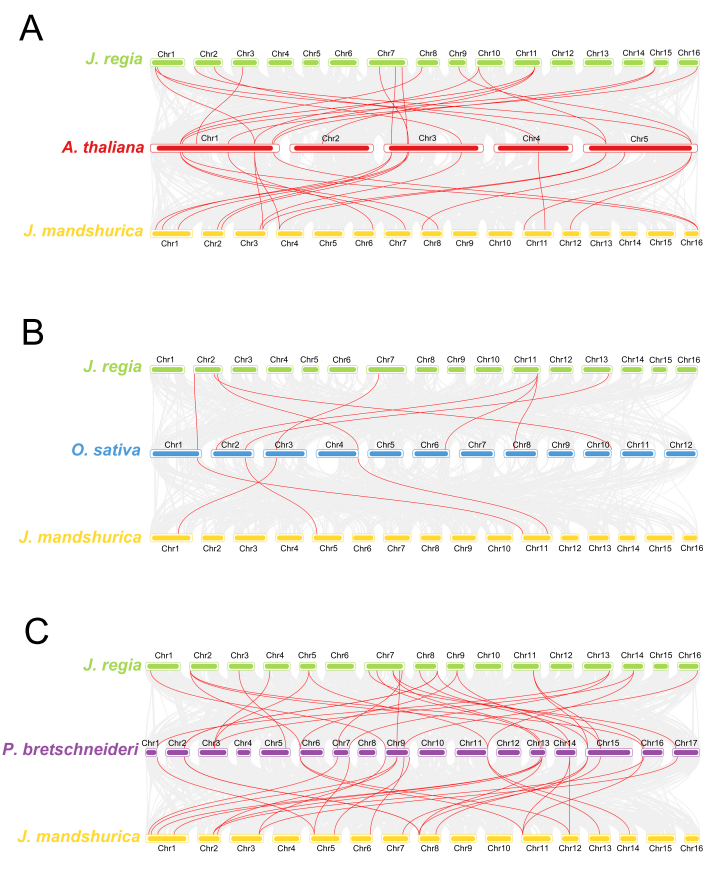


**Figure S4:** Go annotation analysis of *4CLs*. (A) Go annotation of Jr4CLs; (B) Go annotation of Jm4CLs.


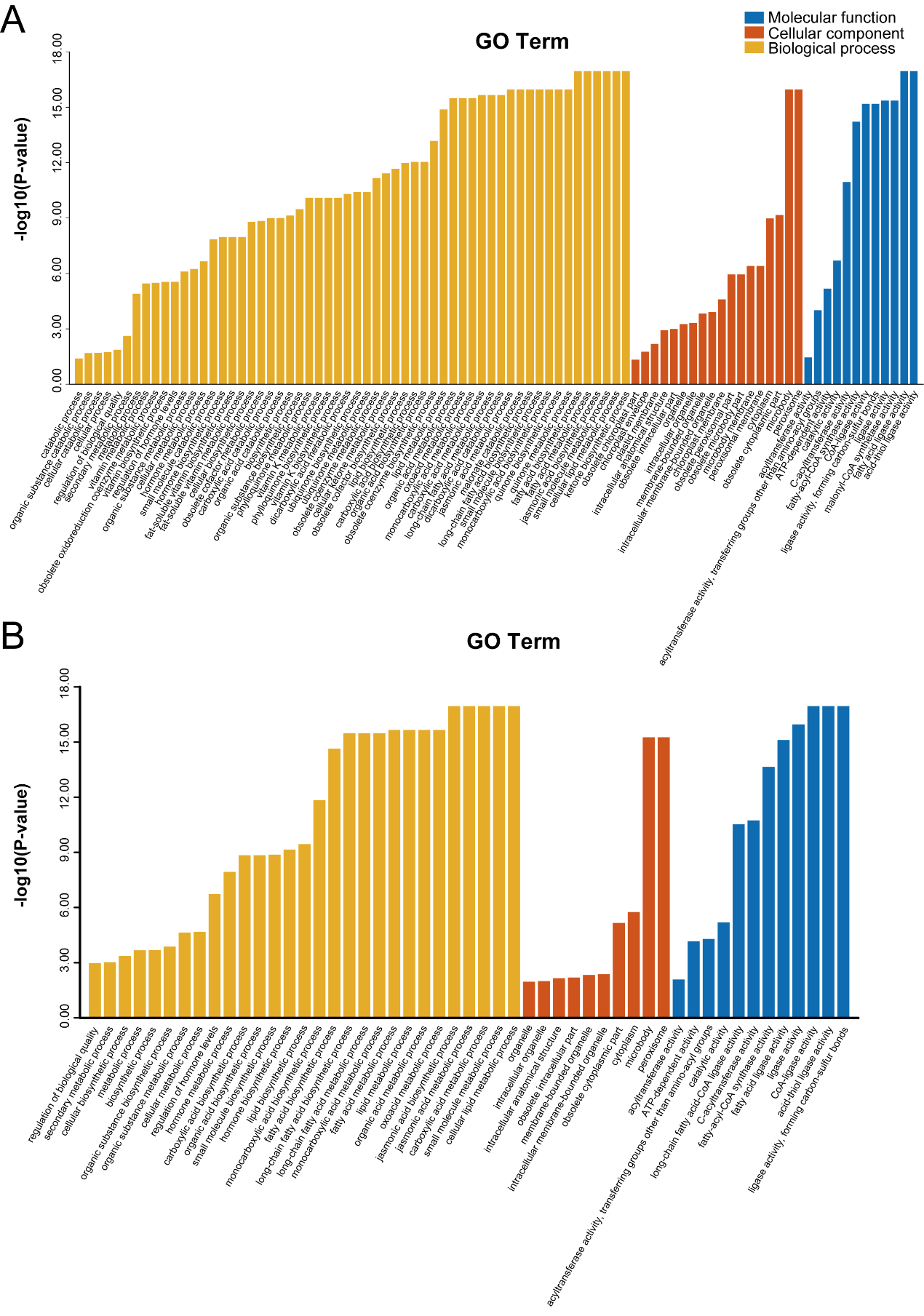


**Figure S5:** Patterns of microRNAs regulate *4CL* genes.


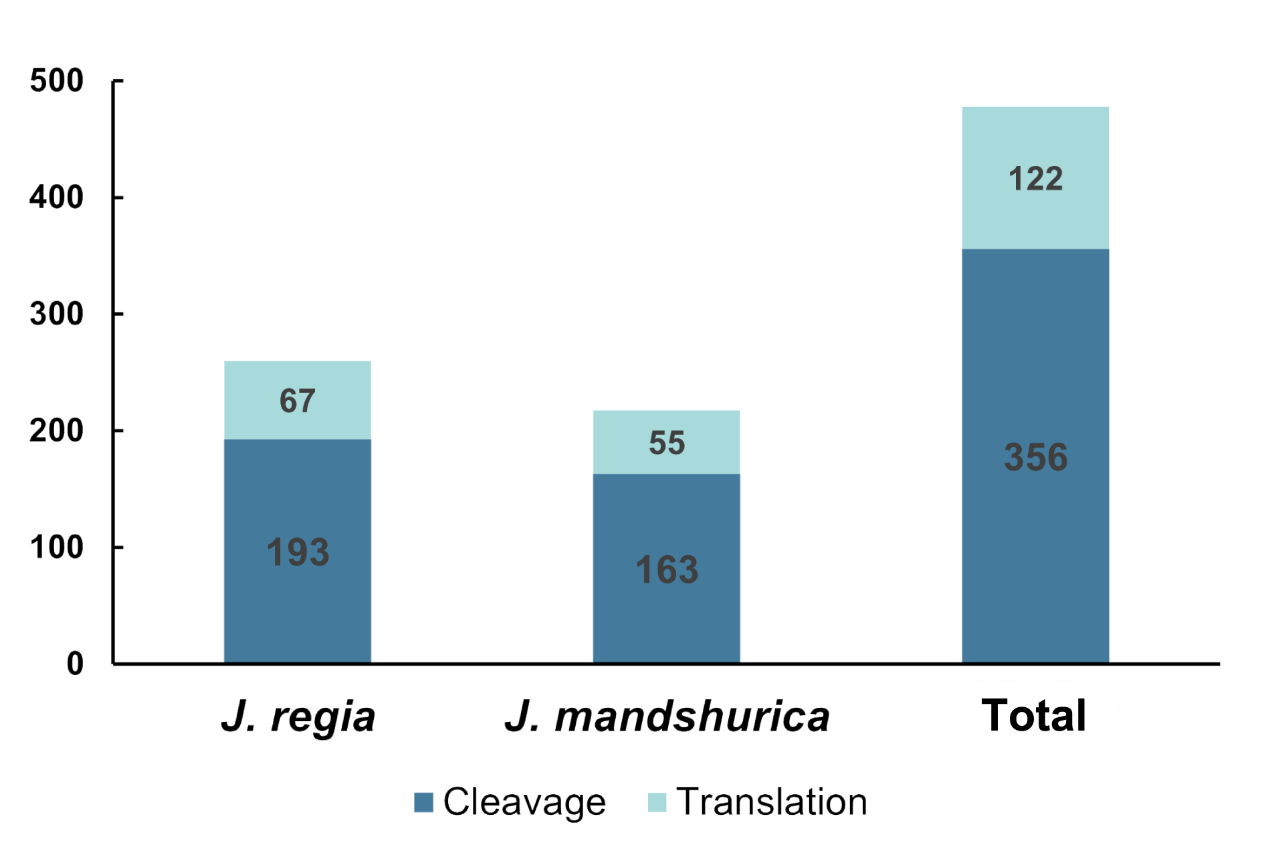


**Figure S6:** The K-means cluster analysis of *Jr4CLs* in infested F26 varieties (A) and in infested F423 varieties (B).


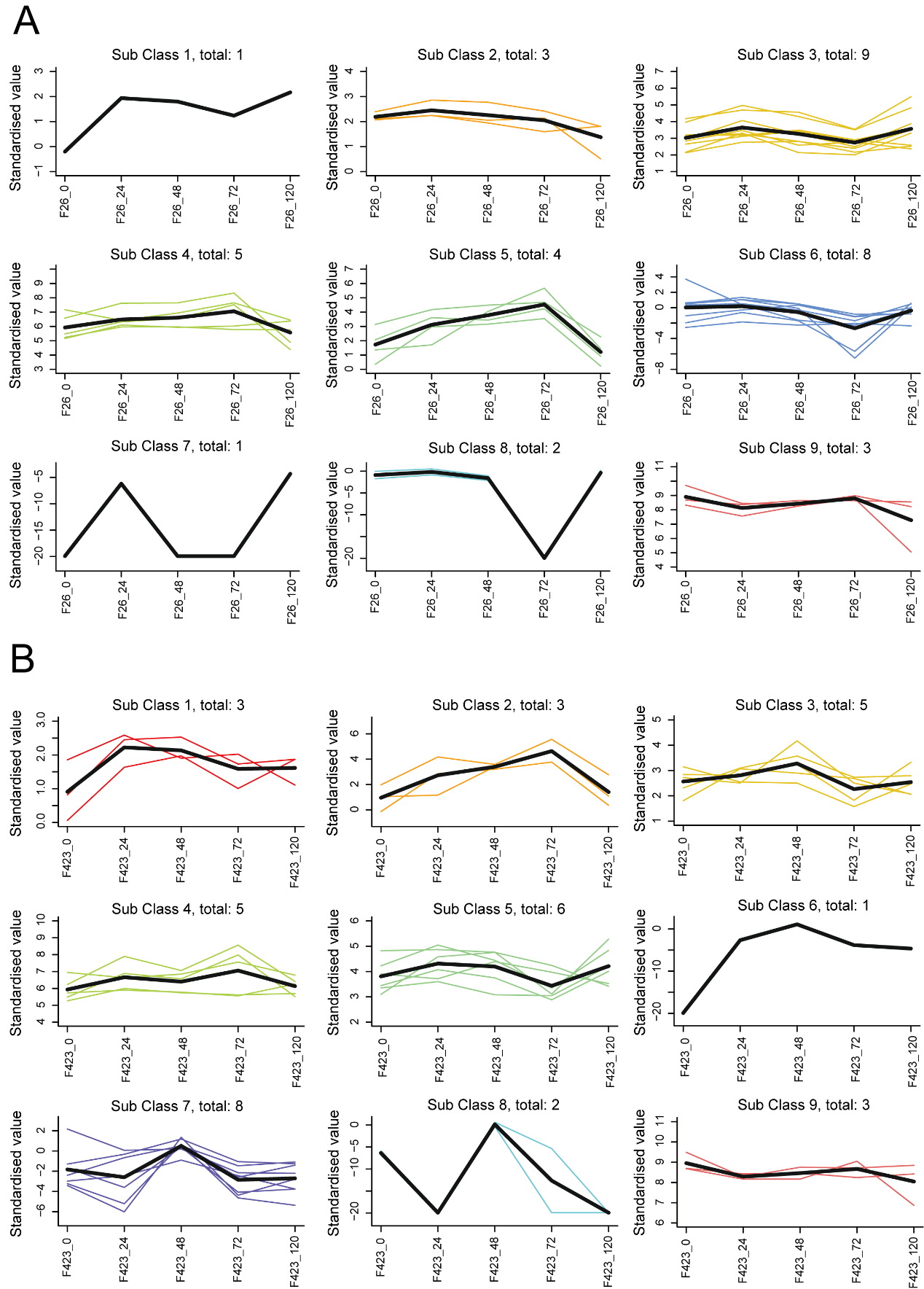


**Figure S7:** The qRT-PCR experiments of *Jr4CLs* and *Jm4CLs* in leaves after salt treatment.


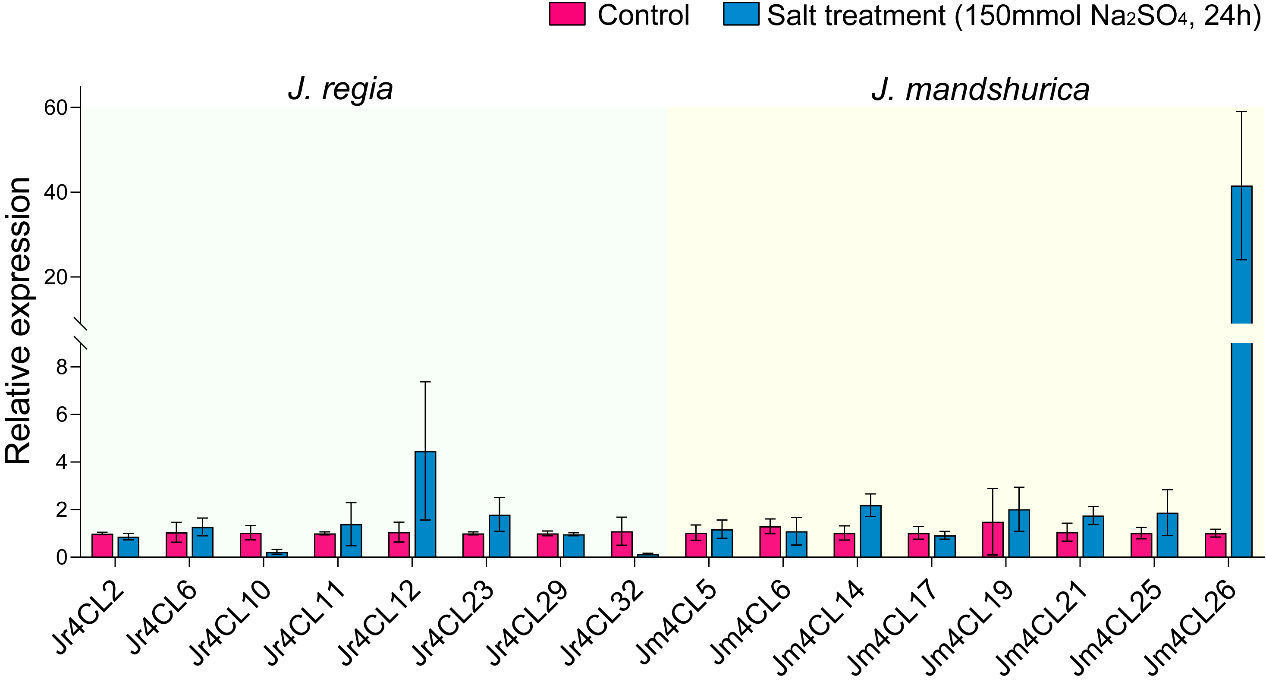


**Figure S8:** Network for *4CL* genes and salt tolerance transcription factors.


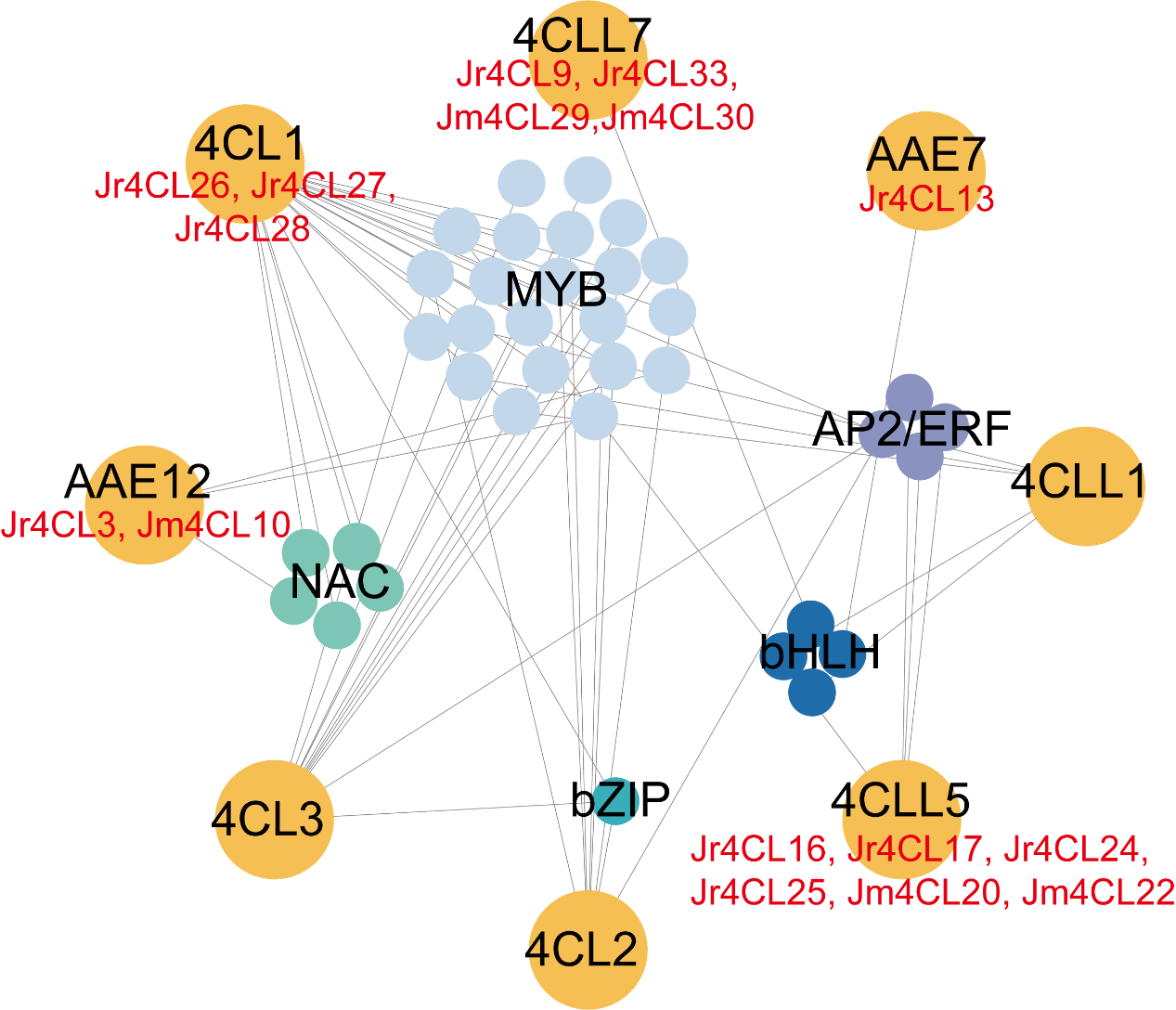

Supplement: Supplementary file 1 — Supplementary Material 1 [file 12870_2024_4899_MOESM1_ESM.docx]
